# Identification of horizontally transferred genes in the genus *Colletotrichum* reveals a steady tempo of bacterial to fungal gene transfer

**DOI:** 10.1186/1471-2164-16-2

**Published:** 2015-01-02

**Authors:** Vinicio D Armijos Jaramillo, Serenella A Sukno, Michael R Thon

**Affiliations:** Departamento de Microbiología y Genética, Universidad de Salamanca, Instituto Hispano-Luso de Investigaciones Agrarias (CIALE), Villamayor, Spain

**Keywords:** Horizontal gene transfer, Phytopathogen, *Colletotrichum*, Anthracnose, Pezizomycotina, Bacteria, Molecular clock

## Abstract

**Background:**

Horizontal gene transfer (HGT) is the stable transmission of genetic material between organisms by means other than vertical inheritance. HGT has an important role in the evolution of prokaryotes but is relatively rare in eukaryotes. HGT has been shown to contribute to virulence in eukaryotic pathogens. We studied the importance of HGT in plant pathogenic fungi by identifying horizontally transferred genes in the genomes of three members of the genus *Colletotrichum*.

**Results:**

We identified eleven HGT events from bacteria into members of the genus *Colletotrichum* or their ancestors. The HGT events include genes involved in amino acid, lipid and sugar metabolism as well as lytic enzymes. Additionally, the putative minimal dates of transference were calculated using a time calibrated phylogenetic tree. This analysis reveals a constant flux of genes from bacteria to fungi throughout the evolution of subphylum Pezizomycotina.

**Conclusions:**

Genes that are typically transferred by HGT are those that are constantly subject to gene duplication and gene loss. The functions of some of these genes suggest roles in niche adaptation and virulence. We found no evidence of a burst of HGT events coinciding with major geological events. In contrast, HGT appears to be a constant, albeit rare phenomenon in the Pezizomycotina, occurring at a steady rate during their evolution.

**Electronic supplementary material:**

The online version of this article (doi:10.1186/1471-2164-16-2) contains supplementary material, which is available to authorized users.

## Background

Horizontal gene transfer (HGT, also called lateral gene transfer) is the stable transmission of genetic material between organisms without the use of vertical inheritance mechanisms, mitosis or meiosis [1]. HGT is common in Bacteria and Archaea and is considered an important force in their evolution [2–5]. In eukaryotes HGT is considered to be rare but an increasing number of studies are reporting HGT events in eukaryotes, and it is now beginning to be considered as an important mechanism of eukaryotic evolution [6]. In fungi, HGT events have been correlated with the gain of pathogenicity traits [7–10] and with a gain of osmotrophic capacity [11, 12]. Genome-wide screens for HGT in fungi have also identified genes related to the metabolism of sugars, nitrogen, amino acids, nucleobases, and macromolecules as well as the acquisition of transporters and secreted proteins [12].

The genetic mechanisms that are responsible for HGT are not well known. The nuclear envelope, the storage of DNA in chromatin, RNA interference systems, separate reproductive cell lines, gene promoter specificity, incompatibility of intron splicing systems, alternative gene codes and others represent barriers against HGT especially in distantly related species (e.g. inter-kingdom HGT) [13–16]. The mechanisms that make HGT possible across distantly related species are not well known. The transference of genetic material from the mitochondrion to the nuclear genome is one possible explanation of ancient HGT from prokaryotes to eukaryotes. Fungi have a large number of plasmids both inside and outside of fungal mitochondria [17], and have been implicated as the vector for this type of transfer [1]. Transposons and viruses are also candidate vectors of HGT. These elements have the potential to transfer genetic material among distantly related species but only in a few cases is there strong evidence to support this type of transfer [1]. Another mechanism is phagocytosis, the “you are what you eat” hypothesis which proposes that the predator–prey relationship could increase the chances of an HGT event in microorganisms [18]. Beyond the vectors needed to achieve HGT, the ecological association of fungi with living and dead organisms may increase the chance of transmitting genetic material laterally [19–21].

A wide range of methods have been proposed to detect HGT including phylogenetic analysis, and the detection of bias in nucleotide composition and codon usage, using naïve Bayes classifiers, correspondence analysis, or Akaike information criterion clustering [22]. Each method has its own strengths and weaknesses but in the case of ancient HGT events, phylogenetic approaches have more power to detect HGT and in general it is considered the most robust analysis method [23].

Among the plant diseases caused by fungi, anthracnose caused by members of the fungal genus *Colletotrichum*, is one of the most destructive, causing significant crop losses worldwide [24–26]. *Colletotrichum* fungi are important as experimental models in studies of many aspects of plant disease [27–30]. Draft genome sequences are now available for several species of *Colletotrichum* and are providing new insight into the study of plant-fungal interactions, , including the evolution of pathogenicity [30–32]. Previously, we identified a secreted protease called CPLS (*Colletotrichum* plant-like subtilisin) that was laterally transferred from plants to an ancestor of *Colletotrichum* [33]. In the present study, we surveyed the genome sequences of three members of the genus *Colletotrichum* to identify additional evidence of HGT to determine the impact of HGT on the evolution of pathogenicity in filamentous fungi. We discuss the potential role of the candidates in pathogenicity and niche adaptation. We found that genes typically transferred by HGT are those that belong to families that are subject to constant gene duplication and loss. We also determined the age of the HGT events, by means of a time calibrated phylogeny, and discuss the timing of HGT events within the context of major geological events. This is the first time that the impact of HGT has been evaluated on a genome-wide scale in *Colletotrichum,* and gives us insight into the evolution of this important genus.

## Results

### Identification of HGT candidates

The most robust method to detect HGT is phylogenetic analysis [23]. Therefore, to detect putative HGT events in three *Colletotrichum* species (*C. graminicola* M1.001 [30], *C. higginsianum* IMI 349063 [30] and *C. gloeosporioides* Cg-14 [32]), we developed a pipeline that consists of a series of BLAST searches and automated filtering designed to reduce the number of unlikely HGT candidates, followed by manual evaluation of phylogenetic trees (Additional file 1: Figure S1). Since our pipeline includes several steps of manual tree inspection, we included several filters designed to reduce the number of candidates that require manual inspection and to limit them to those with the highest sequence similarity. The first BLAST search was performed using a database of proteins from organisms with complete proteome available in UniProt (http://www.uniprot.org). Next, we selected proteins that having at least 80% of the top 120 hits (e-value e-5) with a taxonomic classification other than fungi as candidates for further analysis. The threshold of 80% was selected by evaluating previously described HGT candidates reported by Richards et al. [12], Schmitt and Lumbsch [21] and Richards [28] (see Methods for details). Next, we subjected the HGT candidates to three phylogenetic analyses using different sets of homologous sequences in each phylogeny. The first phylogeny was constructed with homologous sequences from the UniProt complete proteome database (476 proteins from the 3 species were selected). For the second phylogeny we performed a BLAST search of the GenBank nr database and included the 20 best hits from each kingdom (Archaea, Bacteria and Eukaryota) to avoid a possible under or overrepresentation for the abundance of sequences from any one kingdom in the BLAST results. This procedure was performed to observe events of inter-kingdom HGT and to observe the place into the tree of sequences with the same taxonomic label of the query but that were not the best hits in the BLAST search and therefore excluded from the first analysis that uses the best 120 hits. The third phylogeny was constructed with the best 100 BLAST hits from the nr database. The phylogenetic trees were evaluated manually, selecting only those that have well-supported topologies that are clearly incongruent with known species relationships among the taxa. Asymmetric or ladder-shape trees were excluded because such tree topologies are often a signal of long branch attraction or lack of phylogenic information [29]. Additionally, candidates with few homologues or with low sequence similarity to all of their BLAST hits were also excluded. In cases where only hits from two kingdoms were obtained (i.e. Bacteria and Eukaryota), high sequence similarity (a minimum of 30% pairwise similarity) and coverage (over 80% coverage) were required to consider them as candidates. The BLAST searches and tree evaluations were performed serially rather than in parallel to minimize the number of manual phylogenetic tree evaluations required (Additional file 1: Figure S1).

During our analysis, we identified several proteins from *C. gloeosporioides* that appeared to be bacterial-fungal HGT events, had no homology to proteins in the other *Colletotrichum* spp nor to any other fungus. We examined the genomic contigs encoding these proteins and determined they are generally short contigs encoding only one gene. We used MEGABLAST to search the nr database (online version 07-04-2014) using the contig sequences as queries. We found that the contigs align to the *Bacillus pumilus* genome over regions ranging from 203 to 1615 bp and sequence similarities ranging from 87.3% to 92.5% identity (e-values: 0). Based on these BLAST searches, we concluded that these contigs are the result of bacterial contamination of the *C. gloeosporioides* genome assembly and we removed the corresponding HGT candidates from further analysis. We performed another set of BLAST searches to identify homologs to the HGT candidates in the three *Colletotrichum* genomes and then arranged these additional proteins and the HGT candidates into groups of homologous sequences. One group of homologous sequences is comprised of two subtilisin-like serine proteases putatively transferred from a plant ancestor and which we previously described as CPLSs (*Colletotrichum* Plant-Like Subtilisin) [33]. The remaining groups appear to be of bacterial origin. The result was a list of 11 groups of homologous sequences representing 11 HGT events (Table 1). Homologs to HGT4 and HGT5 were described as HGT candidates by Sun et al. [10] and Marcet-Houben and Gabaldón [34] respectively.

**Table 1:** **Summary of tests applied to the final set of HGT candidates**

HGT candidate	Number of homologous sequences used ^a^	BLAST identity threshold	BLAST coverage cut-off	Topology changes with different number of sequences ^b^	Topology changes after editing alignment	Best model	Topology tests (supporting HGT) ^c^	Similarities between ML and BI tree reconstruction (%)
**HGT1**	44	40	90	No	No	LG + I + G + F	ELW, SH	83
**HGT2**	42	35	85	Yes	No	LG + G + F	n.a.	92
**HGT3**	54	41	96	Yes	No	LG + G	ELW	100
**HGT4**	43	40	90	No	No	LG + G + F	n.a.	93
**HGT5**	53	54	97	No	No	LG+G+F	n.a.	96
**HGT6**	46	44	90	Yes	No	WAG + G + F	n.a.	95
**HGT7**	37	35	85	No	No	LG + I + G + F	ELW, SH	84
**HGT8**	47	28	92	No	No	LG + I + G + F	ELW, SH	93
**HGT9**	50	37	90	No	No	LG + G + F	ELW, SH	81
**HGT10**	39	30	75	No	No	LG + G + F	ELW, SH	97
**HGT11**	44	30	80	No	No	LG + G + F	ELW, SH	89

^a^All sequences used have less than 10% of ambiguous quartets in TREE-PUZZLE.

^b^Taking into account only the relative position of horizontal transfer group into the donor group. Topology changes of branches that do not affect the HGT candidates are not reported.

^c^ELW = Expected Likelihood Weight, SH = Shimodaira and Hasegawa, n.a = not applicable. The groups with homologous sequence only in bacteria are not suitable for topology tests.

None of the 11 *C. graminicola* HGT candidates of bacterial origin have introns consistent with their prokaryotic origin. Only 26.4% of the genes encoded in the *C. graminicola* genome lack introns and the probability of selecting 11 intronless genes at random from the *C. graminicola* genome is 4.06e^-7^ further supporting the hypothesis that the 11 candidates are of bacterial origin. To corroborate this calculate we performed a Mann–Whitney-Wilcoxon test to determine whether the two samples of genes (the whole genome and the HGT candidates) are the same, with respect to the number of introns in the genes. These two set of genes were significantly different (p ≈ 0), from which we conclude that the 11 HGT candidates have a different intron distribution than the genome.

We considered that the HGT candidates might lack introns because they are members of gene families that typically lack introns. To test this alternative hypothesis, we first identified 75 homologs to the HGT candidates by searching the proteomes for proteins with the same functional annotation as the candidates. We counted the number of introns in the 75 homologs and compared this sample to the intron content of the rest of the genes in the genome using a Mann–Whitney-Wilcoxon test. The 75 gene sample was not significantly different from the rest of the genes in the genome (p = 0.33). Thus, we conclude that the HGT candidates are not from intron-poor gene families. This evidence supports the hypothesis that the lack of introns in the HGT candidates is the consequence of their bacterial origin.

The GC content of horizontally transferred genes can be different than genes within the recipient genome [22]. We used the Mann–Whitney-Wilcoxon test to determine whether there is a difference in GC content between the 11 HGT candidates in *C. graminicola* and the rest of the genes encoded in the genome. We found that the GC content of the HGT candidates is not different from the rest of the genome (p = 0.337).

### Functional annotation of HGT candidates

The putative function and the biochemical pathways of the HGT candidates were deduced with BRENDA (Release 2012.02) [35], KEGG (update 13-12-2012) [36], MetaCyc 18.5 [37], MEROPS 9.1 [38] and CAZy [39] and are summarized in Table 2. Most of the candidates are involved in processes such as carbohydrate metabolism (HGT5, HGT8, HGT9), amino acid metabolism (HGT1, HGT7, HGT11), secondary metabolism (HGT2) or are secreted degrading enzymes (HGT4 and HGT6). All of the candidates are enzymes and except for HGT2 (glutathionylspermidine synthase) all belong to gene families that are also present in vertically transferred genes. For example HGT1 is annotated as an argininosuccinate lyase (EC 4.3.2.1), the enzyme that catalyzes the formation of fumarate and arginine from L-argininosuccinate in the urea cycle. Three other genes in the *C. graminicola* genome share the same annotation.

**Table 2:** ***Colletotrichum*** **HGT candidates, putative annotation and EC code**

HGT candidate	Locus ID			Annotation	EC code
	***C. graminicola***	***C. higginsianum***	***C. gloeosporioides***		
**HGT1**	GLRG_01134	CH063_01794	CGSP_11293	Argininosuccinate lyase	EC 4.3.2.1
**HGT2**	GLRG_11091 GLRG_11966	CH063_02340 CH063_10640	CGSP_05635	Glutathionylspermidine synthase	EC 6.3.1.8
**HGT3**		CH063_08062	CGSP_09354	Hydroxlacyl-CoA dehydrogenase	EC 1.1.1.35
**HGT4**	GLRG_09635	CH063_05456	CGSP_09262	Oligoxyloglucan reducing-end-specific cellobiohydrolase	EC 3.2.1.150
**HGT5**	GLRG_01139	CH063_01625	CGSP_03952	Glucarate dehydratase	EC 4.2.1.40
**HGT6**	GLRG_11936	CH063_03876	CGSP_08577	Serine endopeptidase S1	
**HGT7**	GLRG_08267		CGSP_12610	L-asparaginase	EC 3.5.1.1
**HGT8**	GLRG_06163		CGSP_01306	Acetyl-CoA synthetase	EC 6.2.1.1
**HGT9**	GLRG_09591		CGSP_11719	2-deoxy-D-gluconate 3-dehydrogenase	EC 1.1.1.125
**HGT10**	GLRG_11949			Monooxygenase, FAD-binding	
**HGT11**	GLRG_10812	CH063_13530		Succinyl-diaminopimelate desuccinylase	EC:3.5.1.18

Four of the HGT candidates are involved in carbohydrate metabolism. Genes in HGT5 encode glucarate dehydratase, the enzyme that transforms D-glucarate to 5-dehydro-4-deoxy-D-glucarate + H2O in the D-glucarate degradation reaction. Genes in HGT9 encode 2-deoxy-D-gluconate 3-dehydrogenase, which is involved in pentose and glucuronate interconversion. Additionally, HGT8 genes encode acetyl-CoA synthetase, which is involved in glucose biosynthesis and in the biosynthesis of the fatty acids and in the Krebs cycle [40]. Finally, HGT4 encodes oligoxyloglucan reducing-end-specific cellobiohydrolase enzymes (glycoside hydrolases belonging to CAZy family GH74), which putatively breakdown carbohydrates in the plant cell wall [41].

Candidate HGT2 (glutathionylspermidine synthase) is the only HGT candidate that does not have vertically transmitted homologs in the *Colletotrichum* genomes and this may represent the acquisition of a completely new gene family by HGT. This enzyme catalyzes the synthesis of glutathionylspermidine and ADP+ orthophosphate from glutathione and spermidine. This reaction is well understood in trypanosomatid parasites and *E. coli* and some evidence supports the role of glutathionylspermidine synthase in detoxification of redox reactions [42, 43].

At least 4 HGT candidates have clear associations with plant interactions and virulence. Members of HGT4 encode secreted glycoside hydrolases belonging to CAZy family GH74 [39] which degrade cellulose in the plant cell wall and are important for virulence in *Magnaporthe oryzae* and other fungi [41, 44, 45]. Members of HGT9, encoding a short chain dehydrogenase, have significant similarity to virulence factors in the Bacterial Virulence Factors Database (Release 3) and the PHI-Base V3.4 database of proteins with roles in pathogen/host interactions. A homolog of this protein in *Cochliobolous heterostrophus*, OXI1, is required for biosynthesis of the secondary metabolite T-toxin [46] and null mutants of the gene show reduced virulence. Members of HGT8 have strong similarity to SidI from *Aspergillus fumigatus* which plays a role in siderophore biosynthesis and subsequently, virulence [47]. Finally HGT1, a family of arginosuccinate lyase genes, share homology with ARG1 of *Fusarium oxysporum* f. sp. *melonis* [48]. Null mutants in *F. oxysporum* have reduced virulence, linking virulence with arginine biosynthesis.

Many of the HGT candidates belong to functional categories that are described as enriched in ‘volatile’ genes by Wapinsky et al. [49]. Volatile genes are those that evolve by duplication and loss in contrast to uniform (genes with the same copy number in all species) and persistent (genes with at least one copy per species) genes. Only HGT1 coincides with a category enriched in persistent genes (arginine metabolism, urea cycle) (Table 3).

**Table 3:** **Relation among the HGT candidates and the enrichment classes described by Wapinski et al.** [49]

HGT candidate	Function	Wapinsky et al. enrichment classes	Category
**HGT1**	Argininosuccinate lyase	Arginine metabolism, urea cycle	Persistent
**HGT2**	Glutathionylspermidine synthase	Detoxification, stress	Volatile
**HGT3**	Hydroxlacyl-CoA dehydrogenase	Oxidoreductase	Volatile
**HGT4**	Oligoxyloglucan reducing-end-specific cellobiohydrolase	Oxidoreductase, cell wall, extracellular region	Volatile
**HGT5**	Glucarate dehydratase		
**HGT6**	Serine endopeptidase S1	Extracellular region	Volatile
**HGT7**	L-asparaginase	AA metabolism	Volatile
**HGT8**	Acetyl-CoA synthetase		
**HGT9**	2-deoxy-D-gluconate 3-dehydrogenase	Oxidoreductase	Volatile
**HGT10**	Monooxygenase, FAD-binding	Oxidoreductase	Volatile
**HGT11**	Succinyl-diaminopimelate desuccinylase	AA metabolism	Volatile

### HGT candidates are expressed during plant infection

We reasoned that if the HGT candidates have roles in virulence then they should be expressed during infection of the host. Therefore, we used the transcriptome profiling experiments reported by O’Connell et al. [30] to identify differences in the level of expression of the HGT gene candidates from *C. graminicola* and *C. higginsianum* during three stages of infection of their respective hosts, maize and *Arabidopsis*. We also examined the expression of a selection of vertically transmitted homologs to the HGT candidates during the infection process. The transcriptional profiling experiment was conducted at three important time points during the infection process: early (*in vitro* or *in planta* appressoria, a specialized fungal structure used to penetrate into the plant; VA, PA), middle (biotrophic phase; BP) and late (necrotrophic phase; NP). Candidates GLRG_11091 (HGT2) and GLRG_11966 (HGT2) of *C. graminicola* and candidates CH063_02340 (HGT2), CH063_10640 (HGT2) and CH063_01625 (HGT5) of *C. higginsianum* are strongly upregulated at the PA and BP time points which are very early in the infection process, suggesting a role in plant penetration or establishment of infection (Additional file 2: Table S2). The candidates GLRG_11936 (HGT6), GLRG_06163 (HGT8), CH063_01794 (HGT1), CH063_13530 (HGT11) and CH063_05456 (HGT4) are upregulated at the latest stage of infection suggesting roles in nutrient uptake.

### A steady tempo of HGT events in the Pezizomycotina

**Figure 1:**
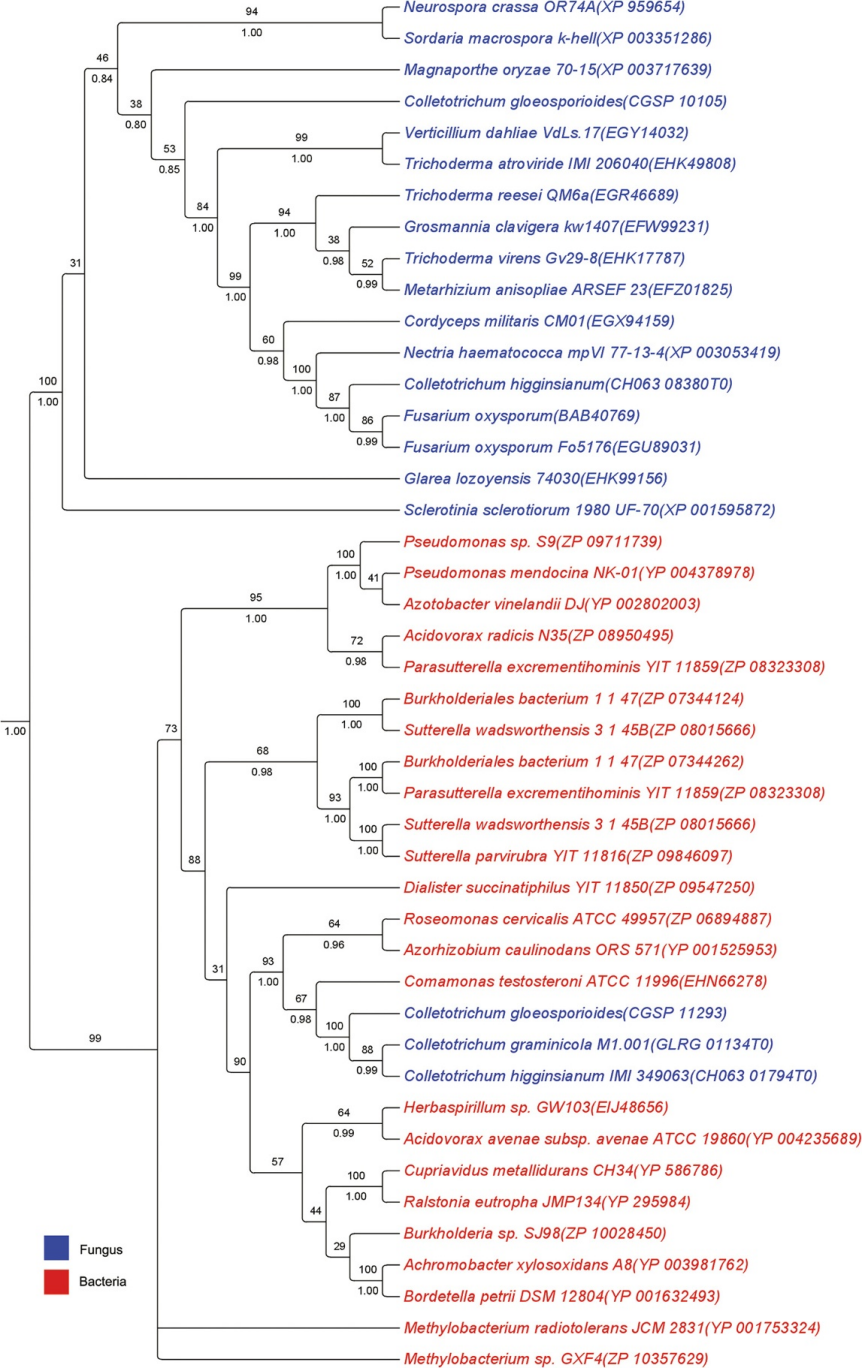
**Ascomycetes species tree and HGT events.** All species that have draft genome sequences and have homologous sequences to the HGT candidates of this study are presented. Black squares indicate the species that contain an HGT candidate. Line width is proportional to bootstrap support. The gray circles indicate the common ancestor of each HGT event and represents the most recent ancestral node where the HGT could have happened. Blue branches represent members of the Pezizomycotina species and yellow branches members of the Saccharomycotina.

**Figure 2:**
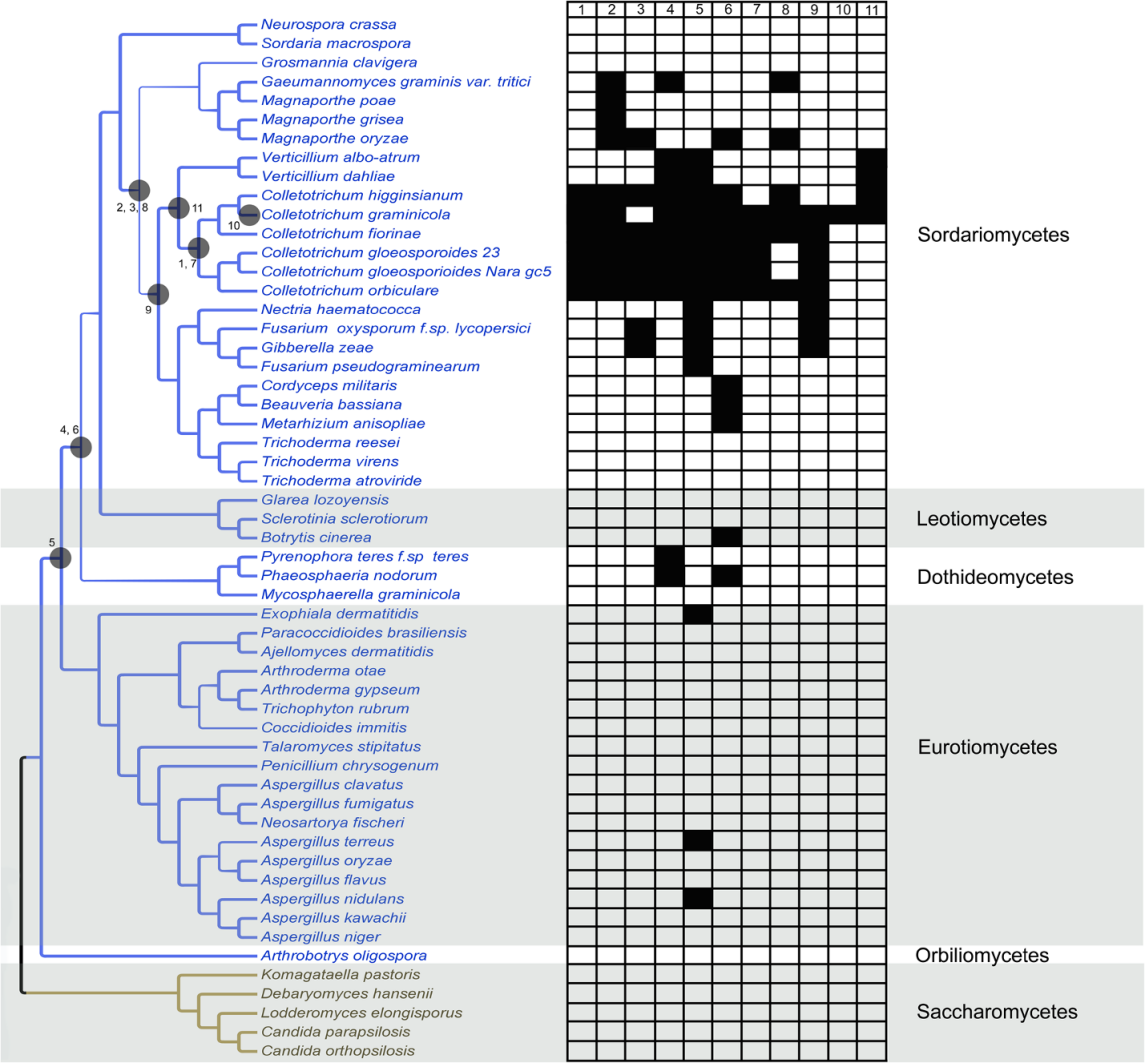
**Time measured phylogeny (millions of years) of some of the important species in this study.** Blue bars are the highest posterior density (HPD) intervals and the values on internal nodes are the median of the time estimation. Grey circles highlight common ancestor of each HGT event. The table shows the HGT events at each node, the median estimated time measured in millions of year and the HPD intervals. Under the figure a time scale shows a schematic representation of geological periods and Eras.

We hypothesized that HGT enables fungi to adopt new ecological niches by giving them access to new nutritional substrates or enabling pathogens or endophytes to jump to new hosts. If this is true then HGT might be more common after major extinction events that may open new niches for colonization by new species. To deduce the age of the HGT events, we reconstructed a species tree with all of the fungal species observed in the HGT detection pipeline. All of the HGT events occurred after the appearance of the Pezizomycotina (Figure 1) and all but three (HGT4, HGT5 and HGT6) occurred after the appearance of the Sordariomycetes. We calculated the approximate divergence times of the lineages using a Bayesian time-measured phylogeny using the fossil *Paleopyrenomycites devonicus* to calibrate the tree [50] (Figure 2). We estimated the minimum ages of transference from bacteria to ancestral members of the Pezizomycotina based on the lower bound of the highest posterior density (HPD) interval. The HGT events occurred over a broad range of geological periods (from the Siluric to the Tertiary). In contrast to our expectations, there is no evidence of a burst of HGT events coinciding with major geological events. In contrast, HGT appears to be a constant, albeit rare phenomenon in the Pezizomycotina, occurring with a steady tempo during their evolution.

While many of the HGT events are ancient, none of them are broadly distributed in extant species of the Pezizomycotina (Figure 1), therefore, many lineages of fungi must have lost the genes. If HGT is associated with niche adaptation then we may find that fungi with certain lifestyles have maintained horizontally transferred genes that are required for the lifestyle.

From Figure 1 we can also estimate the number of gene losses that occurred following the HGT events, based on the absence of orthologs to the HGT candidates in the whole genome sequences that are available in GenBank. We identified the species with presence/absence of each of eleven groups of candidates (Additional file 1: Table S3). 88.72% of Pezizomycotina species (with complete genome available in GenBank) had lost all members of HGT5, 91.73% of species had lost all the members of HGT4 and 93.98% of species had lost all members of HGT6. In contrast, HGT1, HGT2, HGT4, HGT5 and HGT6 are always present in species of the genus *Colletotrichum*. The lifestyle information for each species used in the tree reconstruction was compiled in Additional file 1: Table S4. This information was used to determine if there is a correlation between the lifestyle of the Pezizomycotina species and the presence/absence of the HGT candidates. We did not observe a direct relationship between the lifestyle and the presence of HGT candidates except in HGT4 and HGT6, which are exclusively found in pathogenic species including the entomopathogens *Cordyceps militaris* and *Beauveria bassiana* (in the case of HGT6).

## Discussion

In this study, we developed a semi-automated pipeline to identify HGT candidates in fungal genomes and applied it to three species of the genus *Colletotrichum*. The construction and analysis of phylogenetic trees is recognized as the most reliable method for detecting HGT, so we included several steps of manual phylogenetic trees inspection in the pipeline. Using this pipeline, we detected 12 genes with evidence of HGT from bacteria and one from plants to the genus *Colletotrichum*. The genes were classified into 11 families of homologous sequences (Table 2) representing 11 HGT events. Since our pipeline excludes more distantly related proteins, the HGT events that were identified in this work are only those that yield well-supported phylogenetic trees that pass strict topology tests.

None of the genes of bacterial origin have introns, which is statistically unlikely and consistent with their prokaryotic origin. This observation is consistent with those of other authors who have also reported that horizontally transferred genes from bacteria lack typically introns [51, 52].

Many of the HGT candidates are involved in metabolic processes such as synthesis of amino acids (HGT1, HGT7 and HGT11), lipid metabolism (HGT3), sugar metabolism (HGT8, HGT9 and HGT5) and secondary metabolism (HGT2). HGT1 belongs to a family of argininosuccinate lyase genes responsible for the formation of arginine and fumarate in the urea cycle. In *C. graminicola*, HGT1 member GLRG_01134 as well as the three other arginosuccinate lyase genes in this species are expressed in all stages of the infection process (Additional file 2: Table S2). In contrast, the *C. higginsianum* HGT1 member CH063_01794 is upregulated only late in the infection process. A previous study by Takahara et al. [53] found that arginine biosynthesis in *C. higginsianum* is essential for the early stages of plant infection but the upregulation of CH063_01794 in the last stage of infection (necrotrophy) suggests a different role in this pathogen. In another study, a mutant of *ARG1*, an arginosuccinate lyase encoding gene in *Fusarium oxysporum* f. sp. *melonis* showed reduced virulence [48]. These studies point to an important role of arginine synthesis during the infection process and suggest that the acquisition of an arginosuccinate lyase by HGT may have improved fitness of an ancestral fungal species by increasing virulence.

Other enzymes with roles amino acids metabolism are the members of HGT7, a family of L-asparaginases in the aspartic acid synthesis pathway and HGT11 a family of succinyl-diaminopimelate desuccinylase enzymes in the lysine biosynthesis pathway. HGT7 was found only in the genus *Colletotrichum* and HGT11 is found in *Colletotrichum* as well as the closely related genus *Verticillium*. In bacteria, the horizontal transfer of amino acid metabolic genes have been proposed to provide metabolic plasticity to the recipient species of both non pathogenic [54, 55] and pathogenic [56] species, enabling them to exploit new nutritional sources. This hypothesis has also been proposed for fungal HGT genes [12, 57].

Many of the HGT candidates have functions related to sugar and lipid metabolism (HGT8, HGT9, HGT5 and HGT3), consistent previous reports [12]. Richards et al. [12] propose that the HGT of genes in these functional categories can increase the osmotrophic capacity of fungi. All fungi are osmotrophs, yet horizontally transferred genes are frequently lost in many lineages indicating that there was a subsequent loss of selective pressure to maintain the gene after its initial acquisition. We suggest that HGT is important in niche adaptation and that during the evolution of the fungi, changes in a species’ niche have lead to changes in the selective pressure on the genes required for nutrient uptake.

The putative functions of genes in HGT4 and HGT6 in the degradation of cell wall sugars (HGT4) and proteins (HGT6) and the presence of a predicted secretion signal peptide opens the possibility that they may be secreted lytic enzymes. The time of overexpression of GLRG_11936 (HGT6) in *C. graminicola* (Additional file 2: Table S2) coincides with the necrotrophic phase suggesting that this protein has a bigger role in nutrient acquisition rather than in host penetration. This is also the case with CH063_05456 (HGT4) in *C. higginsianum* which is also overexpressed in the necrotrophic phase. A similar scenario was described by O’ Connell et al. [30] in the transcriptomic analysis of *C. graminicola* and *C. higginsianum*. The authors observed a vast array of lytic enzymes induced at the transition to necrotrophy, the stage at which the pathogen uses dead and dying host cells as a nutrient source to support rapid colonization and sporulation. In contrast, GLRG_09635 (HGT4) of *C. graminicola* does not have changes in expression levels during the infection process, suggesting that its role during infection may be different from that of its *C. higginsianum* counterpart.

The HGT events HGT4 and HGT6 are among the most ancient, having occurred at least 397 million years ago. The transferred genes have been retained in only a few species, all of which are pathogenic, and in the case of HGT6, includes the entomopathogens *Cordyceps militaris* and *Beauveria bassiana*. This suggests that these genes have an important function in host-fungal interactions and that it has maintained this function for millions of years.

A large number of the HGT candidates have functions related to amino acid and carbohydrate metabolism. In the Ascomycetes, gene families with these functions were associated with “volatility”, a term introduced by Wapinsky et al. [49] to describe genes that evolve by duplication and loss in contrast to uniform (genes with the same copy number in all species) and persistent (genes with at least one copy per species) genes. Volatile genes have the ability to evolve by losing or gaining copies without drastic selective consequences, providing new functions or pruning old ones. The functional categories of the HGT candidates reported in the meta analysis of Richards et al. [12] are also rich in volatile functional categories such as carbohydrate metabolism suggesting that HGT is much more likely if the gene is a member of a volatile family.

We also developed a time calibrated phylogenetic tree and deduced the ages of the HGT events (Figure 2). It is important to note that the phylogeny was calibrated with a single fossil and as such, the phylogeny can provide only rough estimates of the dates of the HGT events. Nevertheless, the phylogeny enables us to, for the first time, consider HGT within the context of geologic time. The deduced dates for the HGT events reflect the antiquity of the HGT events reported in this study. Except for HGT10 the transference of all the candidates was situated between 47.68 million years ago (mya) and 424.44 mya (taking into account the mean of the estimations). That reflects very ancient HGT events and despite the high rate of losses in the Pezizomycotina these proteins probably were useful in the adaptation of the organisms to their niches. From the distribution of transfer events in time we can deduce that the HGT occurred all throughout the evolution of Pezizomycotina. Also, given the high percentage of gene losses among the laterally transferred genes we suggest that the number of HGT events detected in the present study represents only a small fraction of the transferences that occurred in the past.

## Conclusions

The phylogenetic evidence presented in this work shows that the genetic flux from bacteria to Pezizomycotina have been constant. Most of the Pezizomycotina with complete genomes available have no evidence of homologs to the *Colletotrichum* HGT candidates, reflecting the propensity of gene loss in horizontally transferred genes. The molecular clock analysis reveals that the HGT events detected in the present have an ancient origin. The HGT candidates typically belong to volatile gene families that are subject to frequent gene duplication and loss. Nevertheless, it is possible that the horizontally transferred gene provided new functions that were useful to ancestral fungi, enabling them to colonize new niches or improve fitness. With the evidence in this work and in other studies, we propose that HGT has been an important evolutionary force in the Pezizomycotina.

## Methods

### Detection of candidates

The first BLAST search was performed using a database of proteins from organisms with complete proteome available in UniProt (downloaded 19-12-2011) (http://www.uniprot.org). Python scripts were used to extract the sequence, description and the two highest taxonomic levels of each sequence. The two highest taxonomic levels of UniProt correspond to kingdom and phylum in Archaea and Bacteria and to superkingdom and kingdom in Eukaryota. The two highest levels were selected to identify the taxonomic assignment at the level of kingdom of the sequences in the database. With this database a BLASTP (v2.2.29+) [58] search (maximum e-value 10^-5^) was performed with all putative proteins predicted in the genomes of *C. graminicola* M 1001*, C. higginsianum* IMI 349063 [30] and *C. gloeosporioides* [32]. We selected proteins that having at least 80% of the top 120 hits (e-value e-5) with a taxonomic classification other than fungi as candidates for further analysis. To choose this threshold, we developed a set of true positive HGT genes by combining the HGT candidates from Richards et al. [12], Schmitt and Lumbsch [21] and Richards [28]. Using the true positive set of proteins, we performed BLAST searches and then constructed phylogenetic trees with the BLAST hits and the query sequence. We evaluated the trees for topologies consistent with HGT of the query sequence. Not all the cases showed HGT patterns after the tree evaluation. 80% was the threshold that enabled us to identify the maximum number of the true positives HGT candidates.

Next, we subjected the HGT candidates to three phylogenetic analyses using different sets of homologous sequences in each phylogeny. The first phylogeny was constructed with homologous sequences from the UniProt complete proteome database (downloaded 19-12-2011) (476 proteins from the 3 species were selected). For the second phylogeny we performed a BLAST search of the GenBank nr database (downloaded 03-02-2012) and included the 20 best hits from each kingdom (Archaea, Bacteria and Eukaryota) to avoid a possible under or overrepresentation for the abundance of sequences from any one kingdom in the BLAST results. The sixty best BLAST hits were aligned using MAFFT [59] and the alignments were edited with Gblocks [60] to remove poorly aligned regions from alignments. The alignments were used to construct phylogenetic trees using PhyML [61] using the default parameters. Each tree was evaluated to identify those with topologies consistent with HGT.

The third phylogeny was constructed with the best 100 BLAST hits from the nr database (downloaded 20-05-2012). The phylogenetic trees constructed as described in the second phylogeny and were evaluated manually, selecting only those that have well-supported topologies that are clearly concruent with HGT. Asymmetric or ladder-shape trees were excluded because such tree topologies are often a signal of long branch attraction or lack of phylogenic information [62]. Additionally, candidates with few homologues or with low sequence similarity to all of their BLAST hits were also excluded. In cases where only hits from two kingdoms were obtained (i.e. Bacteria and Eukaryota), high sequence similarity (a minimum of 30% pairwise similarity) and coverage (over 80% coverage) were required to consider them as candidates. The BLAST searches and tree evaluations were performed serially rather than in parallel to minimize the number of manual phylogenetic tree evaluations required (Additional file 1: Figure S1).

Homologs to the HGT candidates were identified in the *C. graminicola*, *C. higginsianum* and *C. gloeosporioides* genomes using BLAST (v2.2.29+). When no evidence of homology in one or more species was found, we additionally searched the EST database of NCBI and the RNA-seq sequences of O’Connell et al. [30] to find evidence of homologous sequences. To verify the presence of homologous sequences in *C. graminicola*, BLAST searches were performed in the genome of 5 sequenced strains of this species by Rech [63]. Additionally, four newly available *Colletotrichum* genomes were used to verify the absence/presence of each candidate in other members of the genus. This verification was performed through BLAST searches. The genomes used for this purpose were: *Colletotrichum gloeosporioides* 23 (teleomorph, *Glomerella cingulata*) (http://www.jgi.doe.gov), *Colletotrichum fiorinae* MH 18 (teleomorph, *Glomerella acutatum*) (http://www.jgi.doe.gov), *Colletotrichum gloeosporioides* Nara gc5 [31] and *Colletotrichum orbiculare* MAFF 240422 [31].

The 11 groups of candidates were subjected to three tests to ensure that there is sufficient phylogenetic signal to support the HGT hypothesis. For each candidate, we manually inspected the results of the BLAST search to the nr database (downloaded 12-11-2012), selecting appropriate percent identity and coverage thresholds to eliminate distantly related and poorly aligning homologs. Each HGT candidate and its homologs were aligned and analyzed with TREE-PUZZLE [64]. The TREE-PUZZLE algorithm uses a maximum-likelihood approach to resolve the phylogenetic tree of all possible combinations of four sequences in an alignment (quartets). If the maximum likelihood values of the three possible tree topologies of a quartet are too similar, the quartet is labeled as unresolved. Thus, the percentage of unresolved quartets for each sequence can be used as a measure of the phylogenetic signal for that sequence. Using TREE-PUZZLE, we calculated the percentage of unresolved quartets for each sequence and, following the TREE-PUZZLE documentation, discarded those with more than 10% unresolved quartets. Next, we manually edited each multiple sequence alignment, removing regions of low sequence similarity where alignment errors are more likely. A tree was reconstructed for the edited and unedited alignments and the trees were compared to ensure that there was no change in topology due to alignment editing. In no case did we observe a change in topology as a result of alignment editing.

We evaluated the stability of the tree topology when different sets of proteins are used. For each candidate we reconstructed several trees by randomly removing several proteins and comparing the resulting tree topology with that of the original. HGT2, HGT6 and HGT3 had different topologies in this analysis but were stable in the rest of the tests to evaluate the phylogenies.

**Figure 3:**
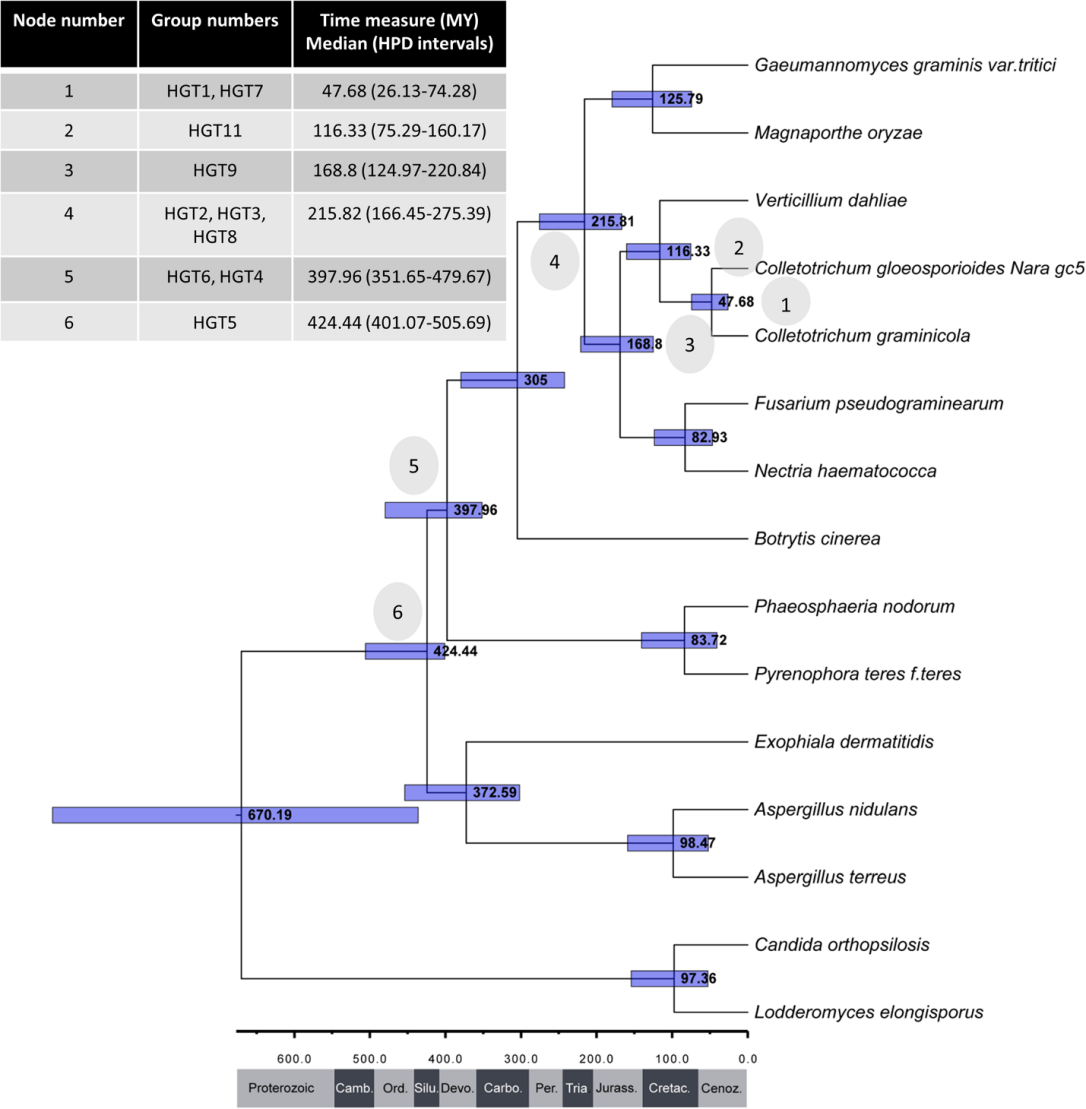
**Maximum likelihood tree of HGT1 (GLRG_01134T0, CH063_01794T0 and CGLO_11293).** The *Colletotrichum* sequences are not located within the fungal lineage, as is expected for vertically inherited genes. Instead, they are clustered within the bacterial lineage. Bootstrap percentages are shown above the branches and posterior probability is shown below the branches when the ML and BI tree topologies coincide. Accession numbers are shown in parenthesis next to each species name.

The data sets were used to perform topology tests to determine whether a tree toplogy supporting vertical inheritance or a star phylogeny are equally well supported by the sequence alignments. Some of the candidates did not have high similarity with fungal sequences or sequences of any other kingdom more than bacteria and in these cases the topology tests could not be performed because the putative HGT sequences could not be constrained with the fungal branch in the tree to evaluate vertical inheritance as an alternative hypothesis. To generate the tree consistent with vertical inheritance, MrBayes [65] was used to constrain all fungal proteins in one branch and the bacterial proteins in other branch. The last topology included in the tests was the star phylogeny. TREE-PUZZLE was used to perform the Expected Likelihood Weight (ELW) test and Shimodaira and Hasegawa (SH) tests. For each candidate with sequence homology in bacteria and fungi the HGT trees were chosen as a better explanation for the data than the tree supporting vertical inheritance or the star phylogeny. Finally, for each alignment, a phylogenetic tree was constructed with PhyML [61] and with MrBayes [65] and the similarity of the two trees was evaluated with T-Coffee [66]. These analyses are summarized in Table 1 and the trees are shown in Figure 3 and Additional file 1: Figures S2-S11. In each case, the HGT candidates form a monophyletic group within the bacterial lineage, indicating a single horizontal transfer event.

To determine whether any of the HGT candidates are mitochondrial genes that were incorrectly assembled into the nuclear genome sequence, or are mitochondrion to nuclear HGT events, we performed a BLASTP (v2.2.29+) search vs all mitochondrial proteins available in the RefSeq database [67]. Proteins of HGT9 show high similarity (e-value 6.17e-22) to protein XP_001875307 a 3-hydroxyacyl-CoA-dehydrogenase (EC 1.1.1.35) from the mitochondrion of *Laccaria bicolor*. However, the most similar BLAST hits are from bacteria (e.g. e-value of 9.33e-116; YP_005460258 from *Actinoplanes missouriensis*). Therefore, we conclude that this is neither a case of mitochondrion to nucleus transfer nor an error in the genome assembly.

To detect signs of contamination of the genome sequences and identify events of HGT of clusters of genes, a BLASTp (v2.2.29+) search of the nr database of NCBI (online version 06-03-2013) was performed with the upstream and downstream proteins of each HGT candidate in the genome of *C. graminicola*, *C. gloeosporioides* and *C. higginsianum*. Most of the neighboring genes showed the expected distribution of BLAST hits with *Colletotrichum* and fungal proteins (Additional file 1: Table S1). Only proteins CH063_08061T0 and CGSP_9355, the flanking genes of members of HGT3 (CH063_08062T0 and CGSP_9355 respectively) have BLAST hits that include bacterial proteins, suggesting the possibility of contamination of the genome sequence with bacterial DNA. However, proteins CH063_08061T0 and CGSP_9355 are orthologous and may have originated from the same HGT event as the members of HGT3 but the phylogenetic evidence was insufficient to conclude this. Additionally, all the candidate proteins were analyzed in the Web server CENSOR [68] to detect repetitive or transposable elements but no evidence of repetitive sequences was found. The entire pipeline is summarized in the Additional file 1: Figure S1.

A maximum posterior tree was constructed with MrBayes, performing 2,000,000 generations of samples, using the substitution matrix and model predicted by MODELGENERATOR but allowing the program to calculate the proportion of invariable sites and the alpha parameter for gamma distribution. Two Multiple Chain Markov Chain Monte Carlo (MC^3^) searches were conducted with four chains each (three heated and one cold). The convergence between them was checked using a sample frequency of 1000 generations. A burn-in of 25% of generations was excluded to reconstruct the Bayesian consensus tree. The topology differences between maximum likelihood and Bayesian trees were quantified with the ratio of identical nodes performed in T-coffee [66]. The percentage of bootstrap and the posterior probability index were joined in the maximum likelihood tree using the program TreeGraph2 [69].

To determine if HGT candidates were the result of contamination a BLASTP (v2.2.29+) (vs the nr database of NCBI) search of the upstream and downstream protein of each candidate in the genome of *C. graminicola*, *C. gloeosporioides* and *C. higginsianum* was performed. This search was done if the neighbor gene was located in the same contig of the candidate, otherwise the analysis was not performed. Additionally, to detect putative mitochondrial to nucleus gene transfers, a BLASTP (v2.2.29+) search against all mitochondrion proteins of RefSeq database [67] was performed. Also all the candidates were submitted to CENSOR [68] to detect the presence of repetitive or transposable elements.

### Functional annotation of HGT candidates

The program Blast2GO 2.8 [70] was used to annotate the HGT candidates with Gene Ontology (GO) terms. The annotations were verified with InterProScan 4 [71] and the putative biochemical functions of candidates were predicted with BRENDA (Release 2012.02) [35], KEGG (update 13-12-2012) [36] and MetaCyc 18.5 [37]. Additionally, MEROPS 9.11 [38] and CAZy [39] databases were explored to further annotate the functions of proteases and carbohydrate active enzymes. To predict the cellular localization of candidates WOLF PSORT v0.2 [72] and SignalP 4.1 server [73] were used.

BLASTP (v2.2.29+) searches of the HGT candidates were performed against PHI-base V3.4 [74], Virulence Factors Database (VFDV) (Release 3) [75] and Database of Fungal Virulence Factors (DFVF) [76] to detect proteins implied in pathogenicity.

The expression data were extracted from O’Connell et al. [30] for *C. graminicola* and *C. higginsianum*. The data is presented in the Additional file 2: Table S2.

### GC content and intron content of the candidates

To determine the presence/absence of introns in HGT candidates a manual inspection of genes was made. To compare the difference of introns content among the candidates and *C. graminicola* a Mann–Whitney-Wilcoxon test was performed using R v2.13.1 [77]. The intron content of each *C. graminicola* gene was calculated from the information available on the Broad institute website (http://www.broadinstitute.org).

The program CodonW [78] was used to calculate the GC content of the whole *Colletotrichum* genome and HGT candidates. These indices were used to calculate the differences of GC content among candidates and whole genomes.

### Species tree reconstruction

To estimate the phylogenetic relationship of the species involved in HGT events, all fungal species found in BLAST searches of the HGT candidates were used (54 species in total). The complete proteome of these species was obtained from the UniProt (http://www.uniprot.org), GenBank (http://www.ncbi.nlm.nih.gov/genbank/), Joint genome institute (http://genome.jgi-psf.org/programs/fungi/index.jsf?projectList) and Broad Institute (http://www.broadinstitute.org/) databases (all databases downloaded 03-05-2013). To reconstruct the species tree, the amino acids inferred from six nuclear genes were chosen. The proteins selected (FG533, FG570, FG832, MS277, MS413 and MS456) from FunyBase [79] demonstrate to be good phylogenetic markers for fungi species trees reconstruction [80] and for that reason these were selected as a query to perform the BLAST searches in the proteomes of the 54 fungal species. Homology was verified making a multiple sequence alignment and tree reconstruction with MAFFT and PhyML respectively to make clear the orthology relationship between taxa. The topological congruence among the protein trees was checked before accepting the protein in the analysis. The six proteins were concatenated for each taxa. The concatenated proteins were aligned with three different programs, MAFFT, MUSCLE [81] and CLUSTALW [82] to evaluate the differences in the phylogenetic reconstruction when different software is used. When the alignment was chosen TrimAl [83], GBLOCKS [60] and Guidance [84] were used to edit it. A tree was reconstructed with each edited and unedited alignment with PhyML with 100 bootstrap repetitions. The best tree was selected by the alignment that produced the tree with the highest bootstrap values. PartitionFinder [85] was used to detect accurate models for the final alignment, using each protein as a partition. The models predicted by PartitionFinder were used in RaxML to calculate the maximum likelihood tree starting with 100 random trees. Finally, a non-parametric bootstrap analysis with 100 replications was performed and the results were summarized in the maximum likelihood tree.

### Molecular clock analysis

To estimate the putative age of the transferred genes a fossil calibrated molecular clock analysis was performed. To avoid problems of convergence in the calculations of the calibrated tree, we selected 15 species from the 54 taxa used in the species tree. The same matrix of 6 concatenated proteins of the species tree was used. The analysis was performed with the BEAST v1.7.5 software package [86]. For each partition (each protein) the LG + I + G model was used. To allow uncorrelated rates of evolution across the tree we use a lognormal relaxed clock model, implementing a Yule process as a tree prior. We use a maximum likelihood tree estimated in PhyML under the LG + I + G model as starting tree. To calibrate the tree we use the estimation of the *Paleopyrenomycites devonicus* fossil age of 400 million years ago (mya) as the lower bound for the Pezizomycotina crown [87]. A lognormal distribution with a mean of 460 (estimated from the results of Lucking et al. [87]), standard deviation of 1 and offset of 400 was used as a prior for the time to the most recent common ancestor (TMRCA) of the Pezizomycotina. Two independent BEAST runs of 15 million generations each were performed. Data was sampled every 1500 generations. The convergence of two runs was visualized with TRACER v1.5 [88] and the Log files and tree files were combined with LogCombiner v1.7.5 [86] dismissing a percentage of the sample in agree with TRACER plots of each run (23.3% for each one). With the remaining trees a maximum clade probability tree was calculated using TreeAnnotator v1.7.5 [86]. The resultant tree was visualized with the FigTree software (http://www.tree.bio.ed.ac.uk/software/figtree/).

## Electronic supplementary material

Additional file 1: **BLAST hits of neighboring genes of HGT candidates.** **Table S3.** Number of gene losses of the HGT in different Pezizomycotina lineages, based on whole genome sequences available in GenBank. **Table S4.** List of the species involved in the HGT events. **Figure S1.** The pipeline used to detect HGT candidates. **Figure S2.** Maximum likelihood tree of HGT2. **Figure S3.** Maximum likelihood tree of HGT3. **Figure S4.** Maximum likelihood tree of HGT4 **Figure S5.** Maximum likelihood tree of HGT5. **Figure S6.** Maximum likelihood tree of HGT6. **Figure S7.** Maximum likelihood tree of HGT7 **Figure S8.** Maximum likelihood tree of HGT8. **Figure S9.** Maximum likelihood tree of HGT9. **Figure S10.** Maximum likelihood tree of HGT10. **Figure S11.** Maximum likelihood tree of HGT11. (PDF 4 MB)

Additional file 2: Table S2: Expression of HGT candidates and homologs in *C. graminicola* and *C. higginsianum*. (XLSX 42 KB)

## Abbreviations

HGT: Horizontal gene transfer
BLAST: Basic Local Alignment Search Tool
GO: Gene Ontology
ELW: Expected Likelihood Weight
SH: Shimodaira and Hasegawa
mya: Million years ago
TMRCA: The most recent common ancestor
HPD: Highest posterior density
VFDV: Virulence Factors Database
DFVF: Database of Fungal Virulence Factors
VA: *in vitro* appressoria
PA: *in planta* appressoria
BP: Biotrophic phase
NP: Necrotrophic phase.
