# Supplementary material for: Identification of horizontally transferred genes in the genus Colletotrichum reveals a steady tempo of bacterial to fungal gene transfer
Source: BMC Genomics. 2015 Jan 2;16(1):2. doi: 10.1186/1471-2164-16-2 (PMC4320630; doi:10.1186/1471-2164-16-2)
Supplement: Supplementary file 1 — Additional file 1: BLAST hits of neighboring genes of HGT candidates. Table S3. Number of gene losses of the HGT in different Pezizomycotina lineages, based on whole genome sequences available in GenBank. Table S4. List of the species involved in the HGT events. Figure S1. The pipeline used to detect HGT candidates. Figure S2. Maximum likelihood tree of HGT2. Figure S3. Maximum likelihood tree of HGT3. Figure S4. Maximum likelihood tree of HGT4 Figure S5. Maximum likelihood tree of HGT5. Figure S6. Maximum likelihood tree of HGT6. Figure S7. Maximum likelihood tree of HGT7 Figure S8. Maximum likelihood tree of HGT8. Figure S9. Maximum likelihood tree of HGT9. Figure S10. Maximum likelihood tree of HGT10. Figure S11. Maximum likelihood tree of HGT11. (PDF 4 MB) [file 12864_2014_6926_MOESM1_ESM.pdf]

Supplementary Tables

Table S1. BLAST hits of neighboring genes of HGT candidates, associated chromosome location, number of introns and GC content of the candidates.

HGT group	Accession numbers			Taxonomic status of top BLAST hits of neighboring genes		Associated chromosome	Number of introns	GC content (%)
	C. graminicola	*C. higginsianum*	*C. gloeosporioides*	Upstream gene	Downstream gene			
HGT1	GLRG_01134			Fungi	Fungi	8	0	56.9
		CH063_01794		Fungi	Fungi	-	0	61.7
			CGSP_11293	Fungi	Fungi	-	0	54.1
HGT2	GLRG_11091			Fungi	*Colletotrichum*	10	0	52.3
	GLRG_11966			-	*C. graminicola*	-	0	50.3
		CH063_02340		-	Fungi	-	0	57.2
		CH063_10640		-	*Colletotrichum*		0	58.9
			CGSP_05635	Fungi	Fungi	-	0	54
HGT3		CH063_08062		Fungi-bacteria	-	-	0	54.5
			CGSP_09354	Fungi	Fungi-bacteria	-	0	52.7
HGT4	GLRG_09635			Fungi	-	2	0	60.4
		CH063_05456		-	*Colletotrichum*	-	0	67.4
			CGSP_09262	Fungi	Fungi	-	0	59.2
HGT5	GLRG_01139			Fungi	*Colletotrichum*	8	0	54.4
		CH063_01625		Fungi	Fungi	-	0	61.4
			CGSP_03952	Fungi	Fungi	-	0	55.8
HGT6	GLRG_11936			-	-	-	0	61.6

		CH063_03876		-	Fungi	-	0	68
			CGSP_08577	Fungi	Fungi	-	0	65.3
	GLRG_08267			Fungi	Fungi	10	0	52.4
**HGT7**			CGSP_12610	Fungi	Fungi	-	0	53
	GLRG_06163			Fungi	Fungi	5	0	57
**HGT8**			CGSP_01306	Fungi	Fungi	-	0	53.4
	GLRG_09591			Fungi	Fungi	2	0	65
**HGT9**			CGSP_11719	Fungi	Fungi	-	0	59.4
**HGT10**	GLRG_11949			-	-	-	0	53.4
	GLRG_10812			Fungi	*Colletotrichum*	6	0	71.3
**HGT11**		CH063_13530		-	*Colletotrichum*	-	0	70.5

Table S3. Number of gene losses of the HGT in different Pezizomycotina lineages, based on whole genome sequences available in GenBank.

**HGT Group**	**Lineage**	**Number of species available in GenBank**	**Losse s**	**Percentage of losses (%)**
HGT1	Colletotrichum	6	0	0
HGT7	Colletotrichum	6	0	0
HGT10	Colletotrichum	6	0	0
HGT11	Colletotrichum/Verticillium	9	1	11.11
HGT2	Sordariomycetes	53	42	79.25
HGT3	Sordariomycetes	53	45	84.91
HGT8	Sordariomycetes	53	47	88.68
HGT9	Sordariomycetes	53	45	84.91
HGT6	Pezizomycotina	133	125	93.98
HGT4	Pezizomycotina	133	122	91.73
HGT5	Pezizomycotina	133	118	88.72

Table S4. List of the species involved in the HGT events, their lifestyles and the presence/absence of the HGT candidates

**Species name**	**Lifestyle**	**HGT 1**	**HGT 2**	**HGT 3**	**HGT 4**	**HGT 5**	**HGT 6**	**HGT 7**	**HGT 8**	**HGT 9**	**HGT1 0**	**HGT11**
Botryotinia fuckeliana	Necrotroph [1]						■					
Verticillium albo-atrum	Necrotroph [1]				■	■						■
Verticillium dahliae	Necrotroph [1]				■	■						■
Gibberella zeae	Necrotroph [1]			■		■				■		
Fusarium pseudograminearum	Necrotroph [2]					■						
Phaeosphaeria nodorum	Necrotroph [1]				■		■					
Pyrenophora teres	Necrotroph [1]				■							
Nectria haematococca	Necrotroph/saprotroph [1]					■				■		
Gaeumannomyces graminis	Necrotroph/saprotroph [3]		■		■				■			
C. orbiculare	Hemibiotroph [4]	■	■	■	■	■	■	■	■	■		■
C. gloeosporioides	Hemibiotroph [4]	■	■	■	■	■	■	■		■		■
C. graminícola	Hemibiotroph [5]	■	■		■	■	■	■	■	■	■	■
C. higginsianum	Hemibiotroph [6]	■	■	■	■	■	■		■			■
Magnaporthe grisea	Hemibiotroph [1]		■									
Metarhizium anisopliae	Hemibiotrophs [7]						■					
Magnaporthe oryzae	Hemibiotrophs [7]		■	■			■		■			
Fusarium oxysporum	Hemibiotroph/ endophyte/saprotroph [1]			■		■				■		
Magnaporthe poae	Plant pathogen [8]		■									
C. fiorinae	Plant pathogen, entomopathogen [9]	■	■	■	■	■	■	■	■	■		■
Cordyceps militaris	Entomopathogenic fungus [10]						■					
Beauveria bassiana	Entomopathogenic fungus [11]						■					
Aspergillus terreus	Soil saprophyte-occasional animal pathogen [12]					■						
Exophiala dermatitidis	Saprophyte, human pathogen [13,					■						

	14]											

Supplementary figures

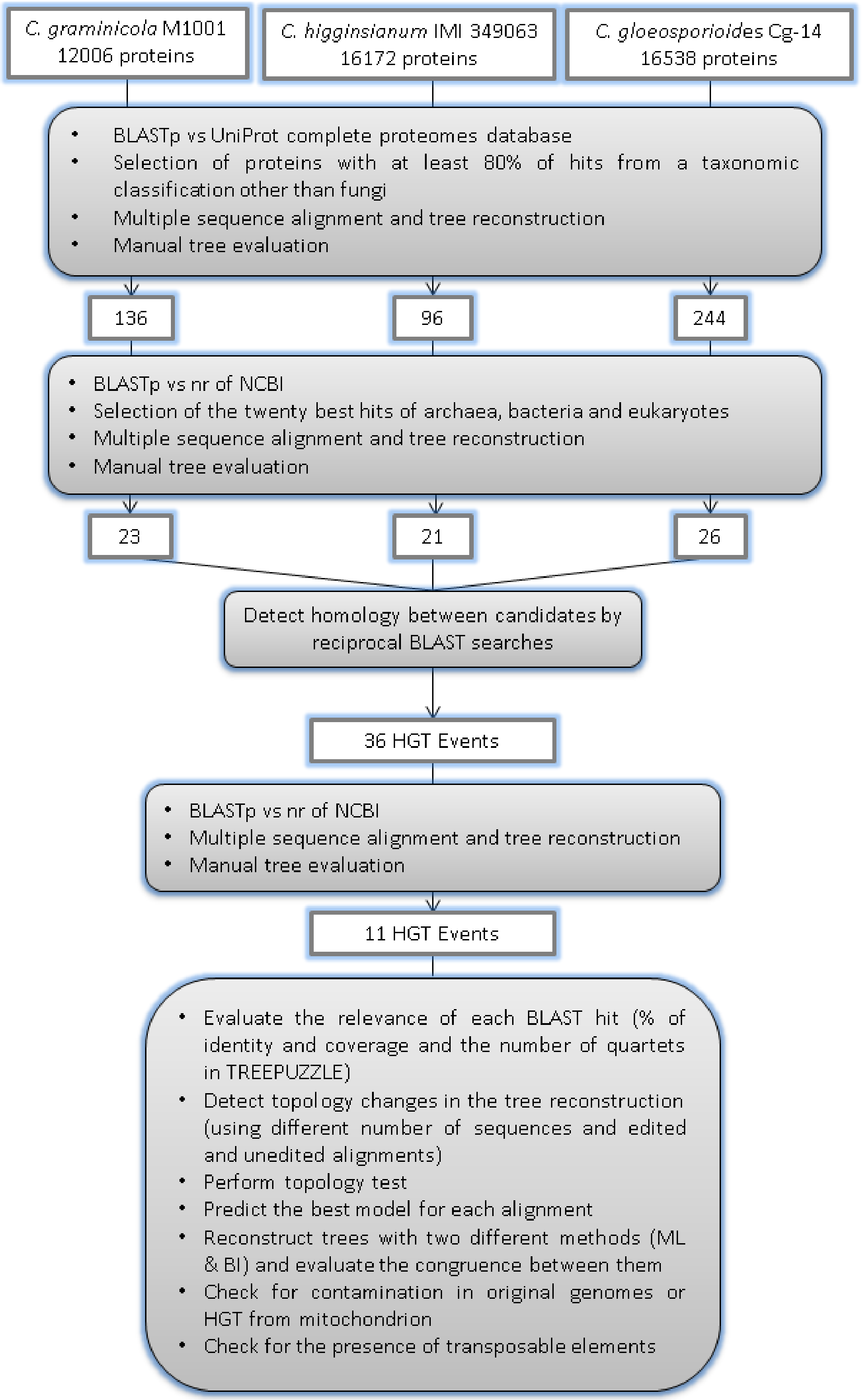

Figure S1. Diagram of the pipeline used in this work to detect HGT candidates in three species of the genus *Colletotrichum*.

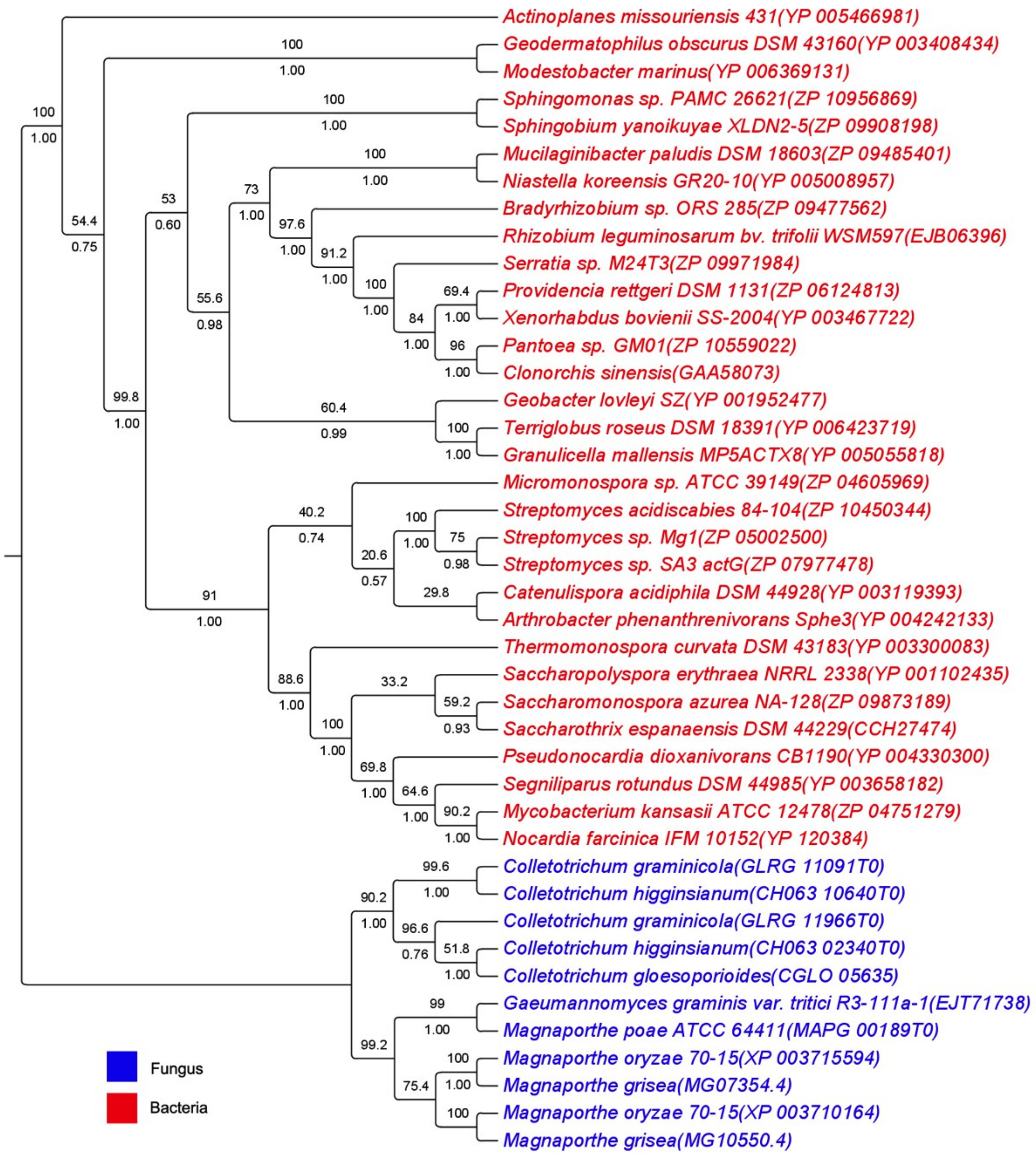

Figure S2. Maximum likelihood tree of HGT2 (GLRG_11091T0, GLRG_11966T0, CH063_02340T0, CH063_10640T0, CGLO_05635). Bootstrap percentage is shown above the branches and posterior probability is shown below the branches. Accession numbers are shown in parenthesis next to the species names.

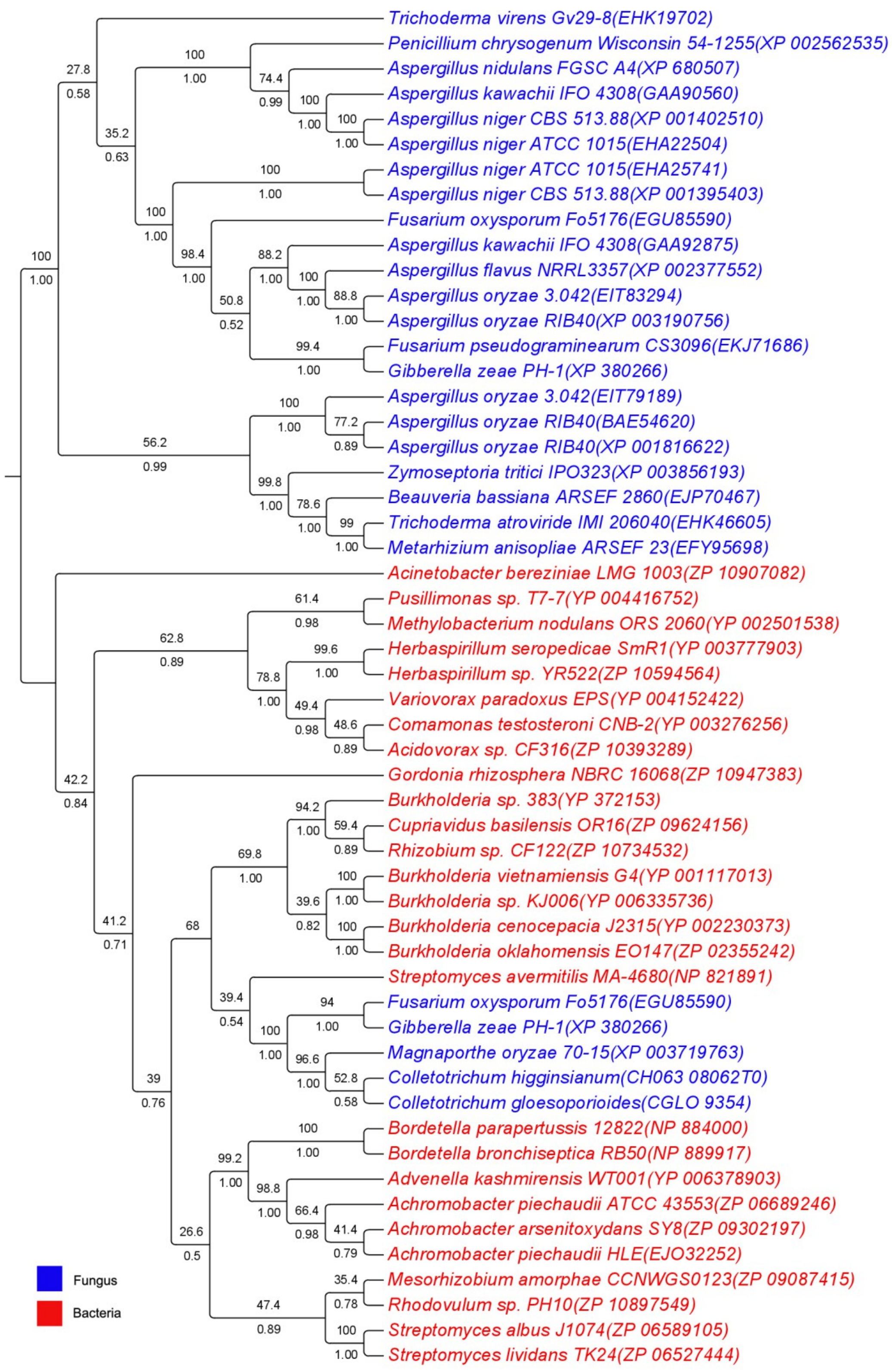

Figure S3. Maximum likelihood tree of HGT3 (CGSP_9354, CH063_08062T0). Bootstrap percentage is shown above the branches and posterior probability is shown below the branches. Accession numbers are shown in parenthesis next to the species names.

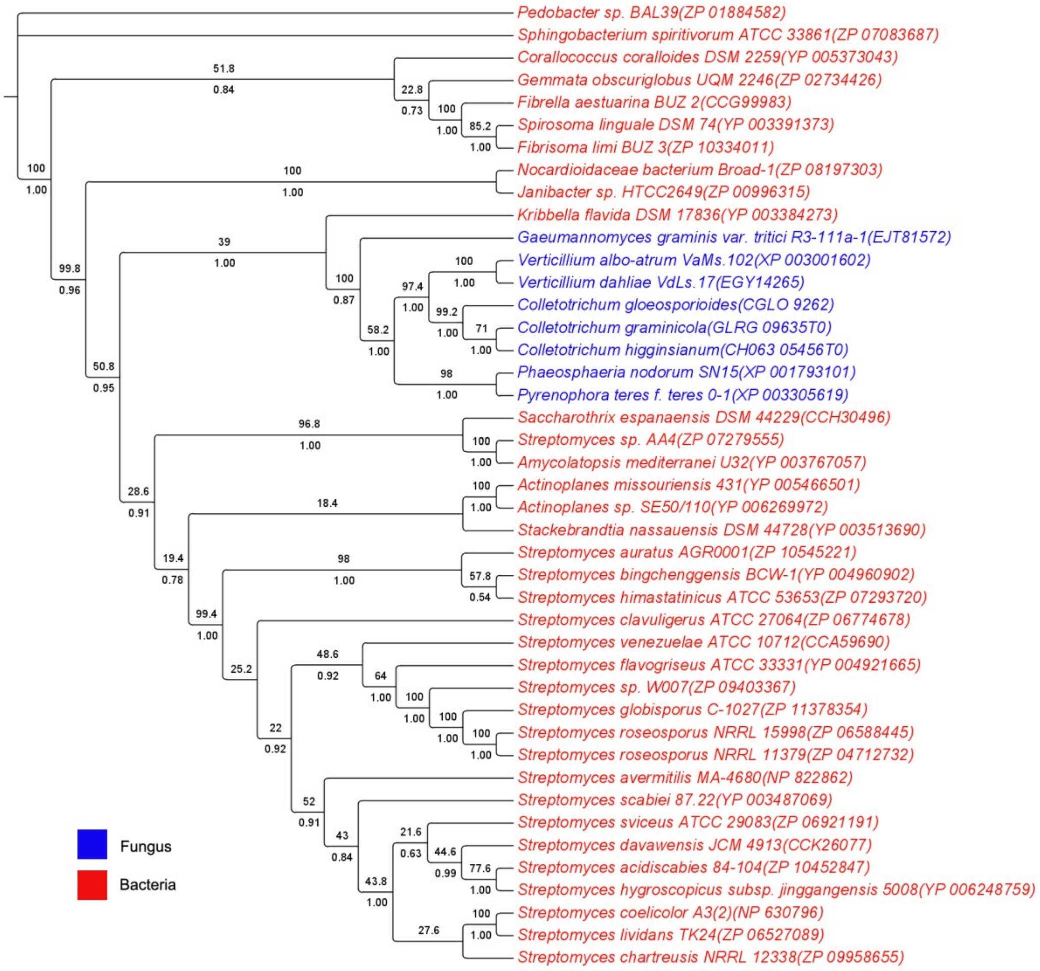

Figure S4. Maximum likelihood tree of HGT4 (CGSP_9262, CH063_05456T0, GLRG_09635T0). Bootstrap percentage is shown above the branches and posterior probability is shown below the branches. Accession numbers are shown in parenthesis next to the species names.

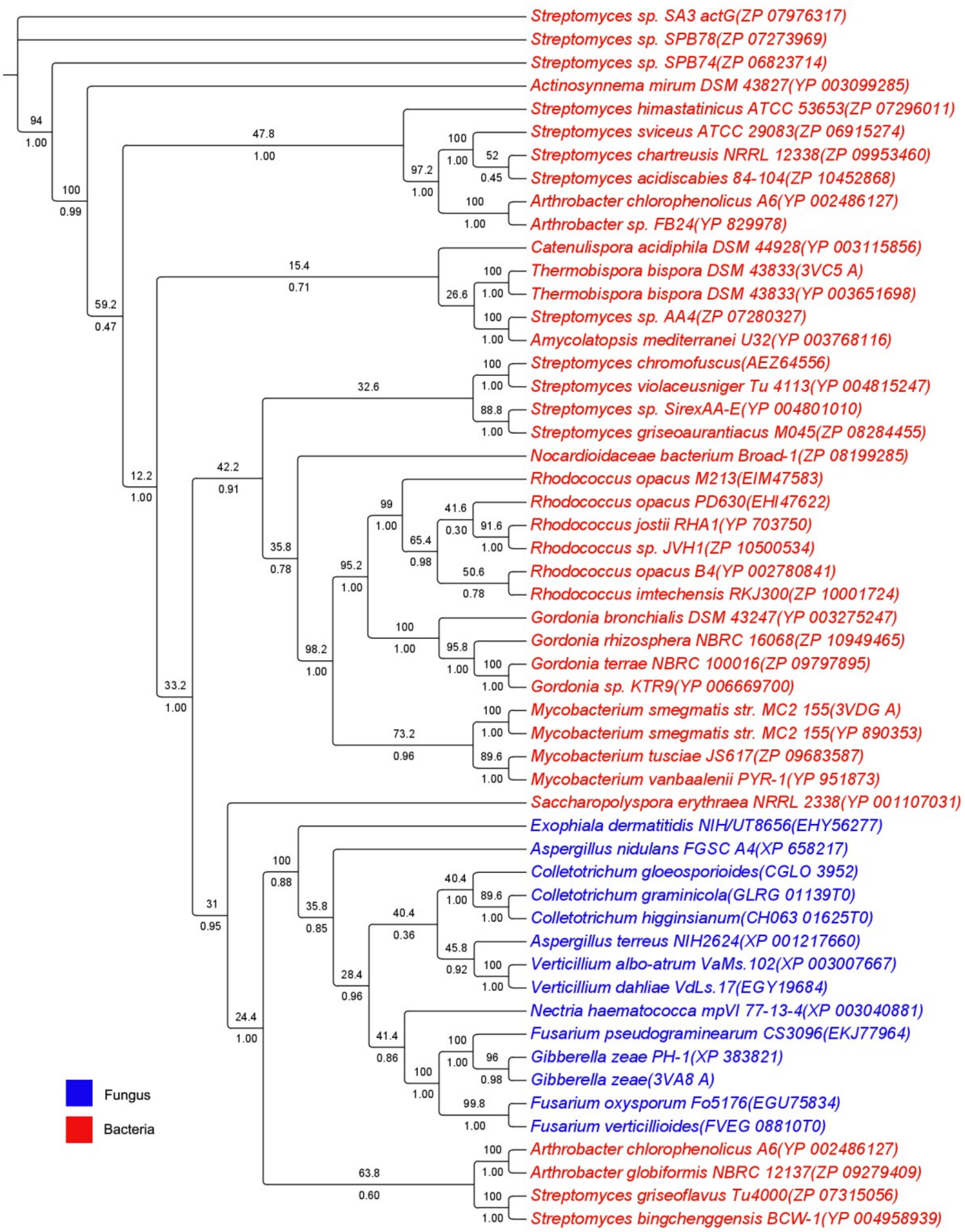

Figure S5. Maximum likelihood tree of HGT5 (CGSP_3952, CH063_01625T0, GLRG_01139T0). Bootstrap percentage is shown above the branches and posterior probability is shown below the branches. Accession numbers are shown in parenthesis next to the species names.

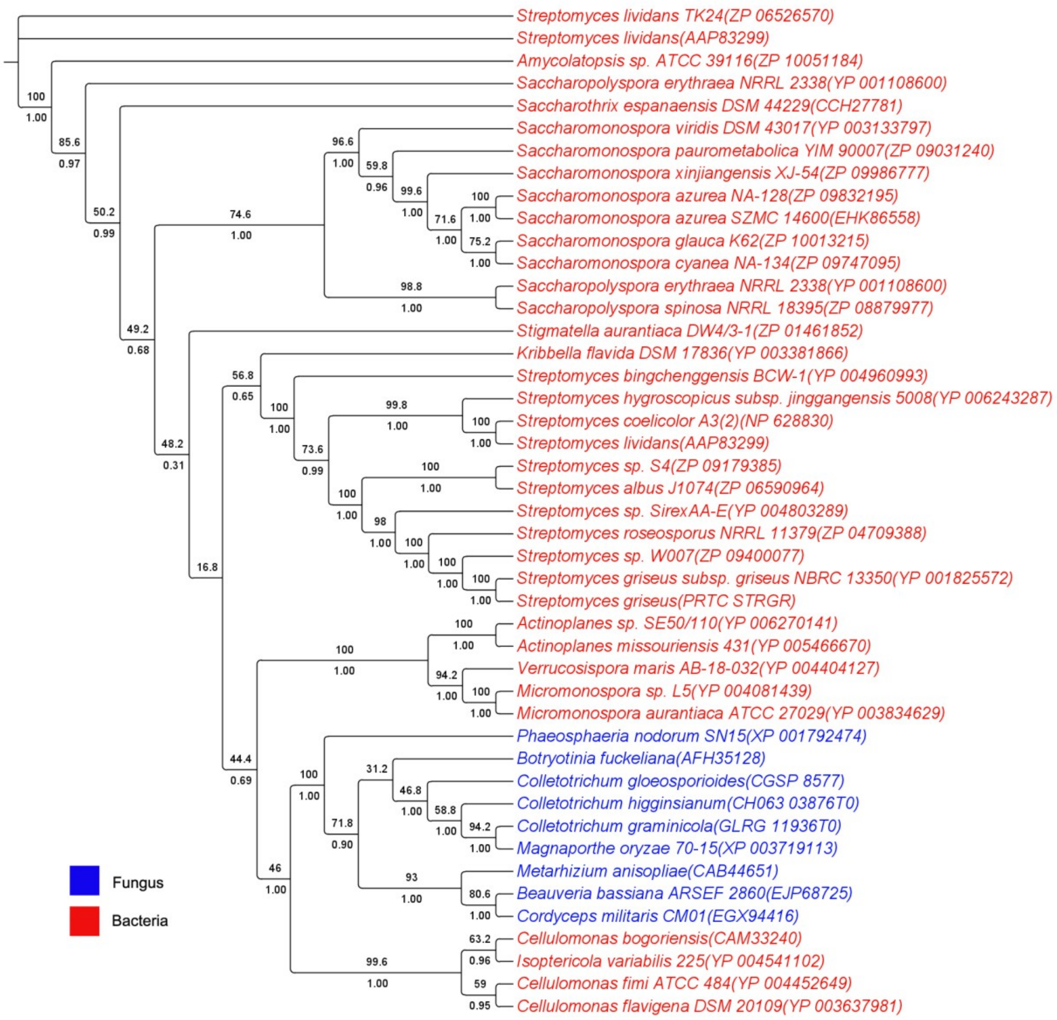

Figure S6. Maximum likelihood tree of HGT6 (CH063_03876T0, CGSP_8577, GLRG_11936T0). Bootstrap percentage is shown above the branches and posterior probability is shown below the branches. Accession numbers are shown in parenthesis next to the species names.

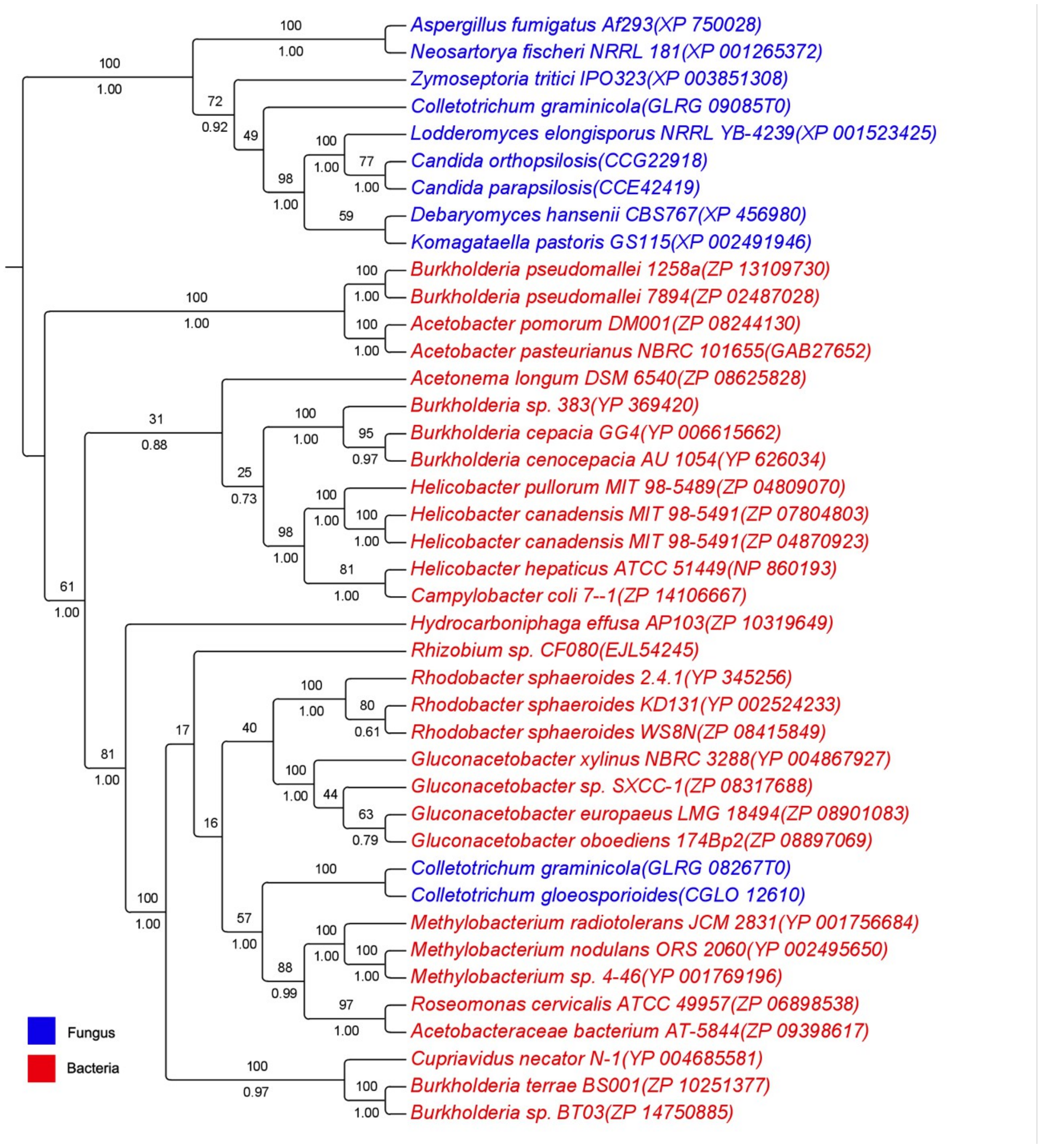

Figure S7. Maximum likelihood tree of HGT7 (CGSP_12610, GLRG_08267T0). Bootstrap percentage is shown above the branches and posterior probability is shown below the branches. Accession numbers are shown in parenthesis next to the species names.

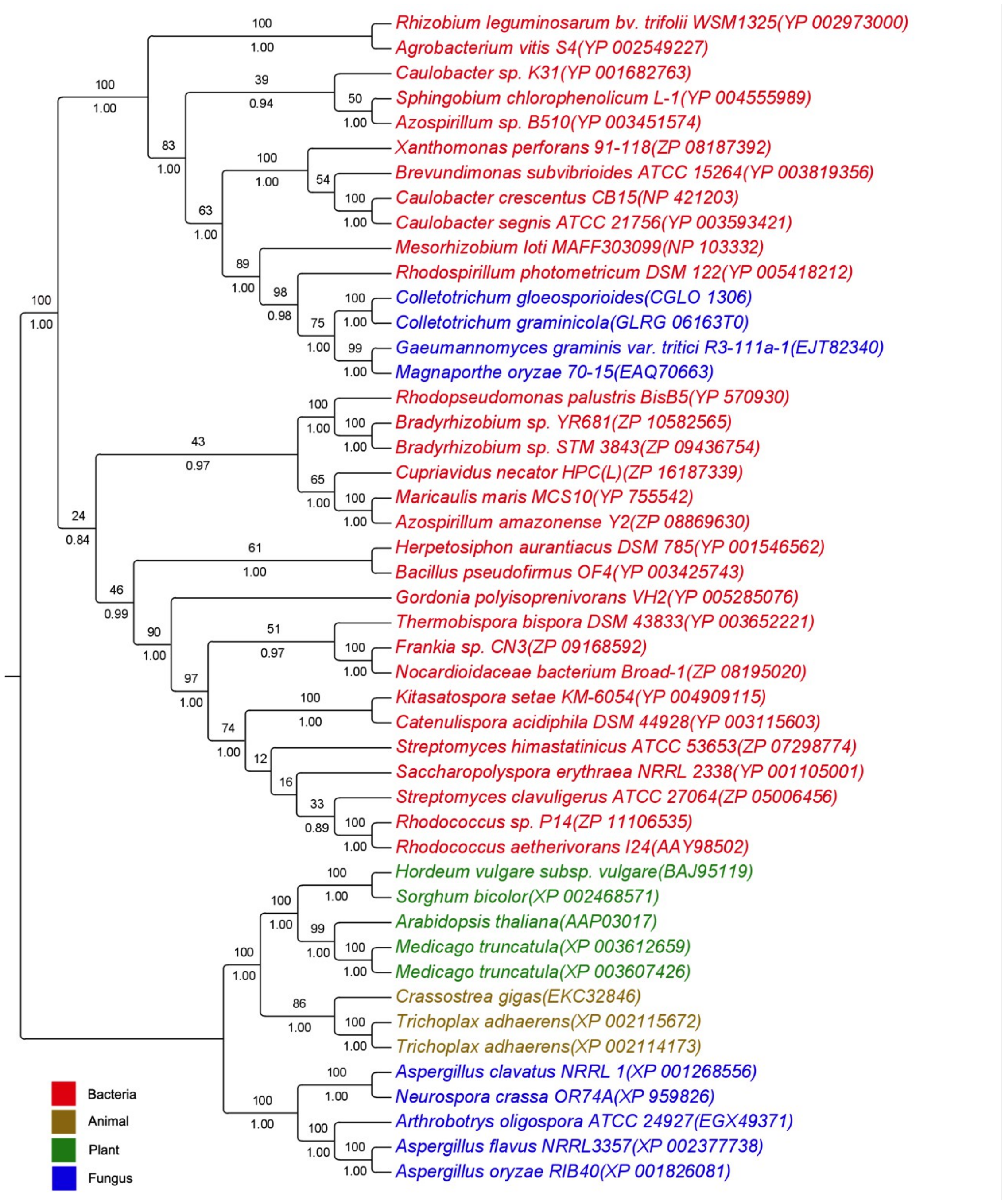

Figure S8. Maximum likelihood tree of HGT8 (CGSP_1306, GLRG_06163T0). Bootstrap percentage is shown above the branches and posterior probability is shown below the branches. Accession numbers are shown in parenthesis next to the species names.

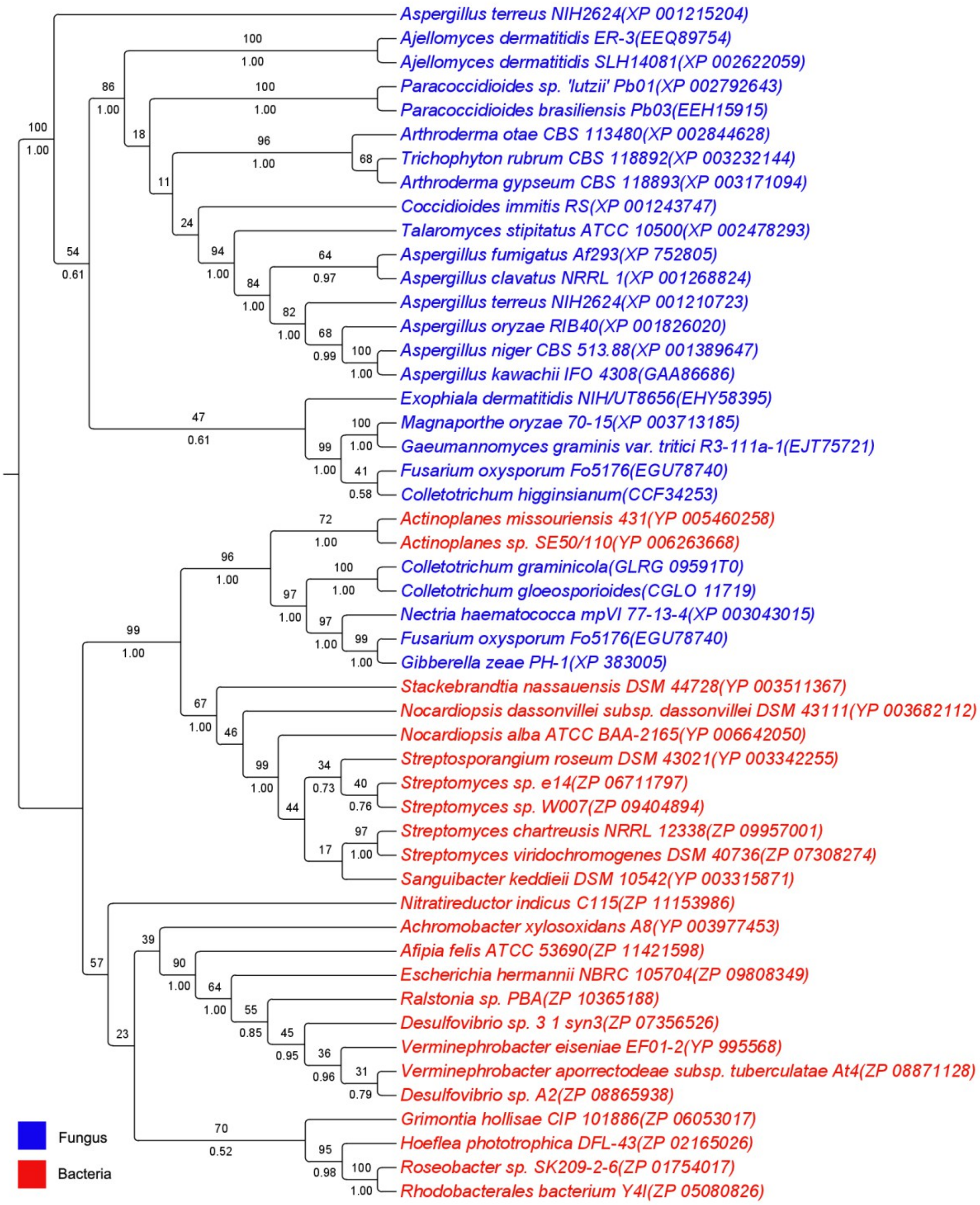

Figure S9. Maximum likelihood tree of HGT9 (CGSP_11719, GLRG_09591T0). Bootstrap percentage is shown above the branches and posterior probability is shown below the branches. Accession numbers are shown in parenthesis next to the species names.

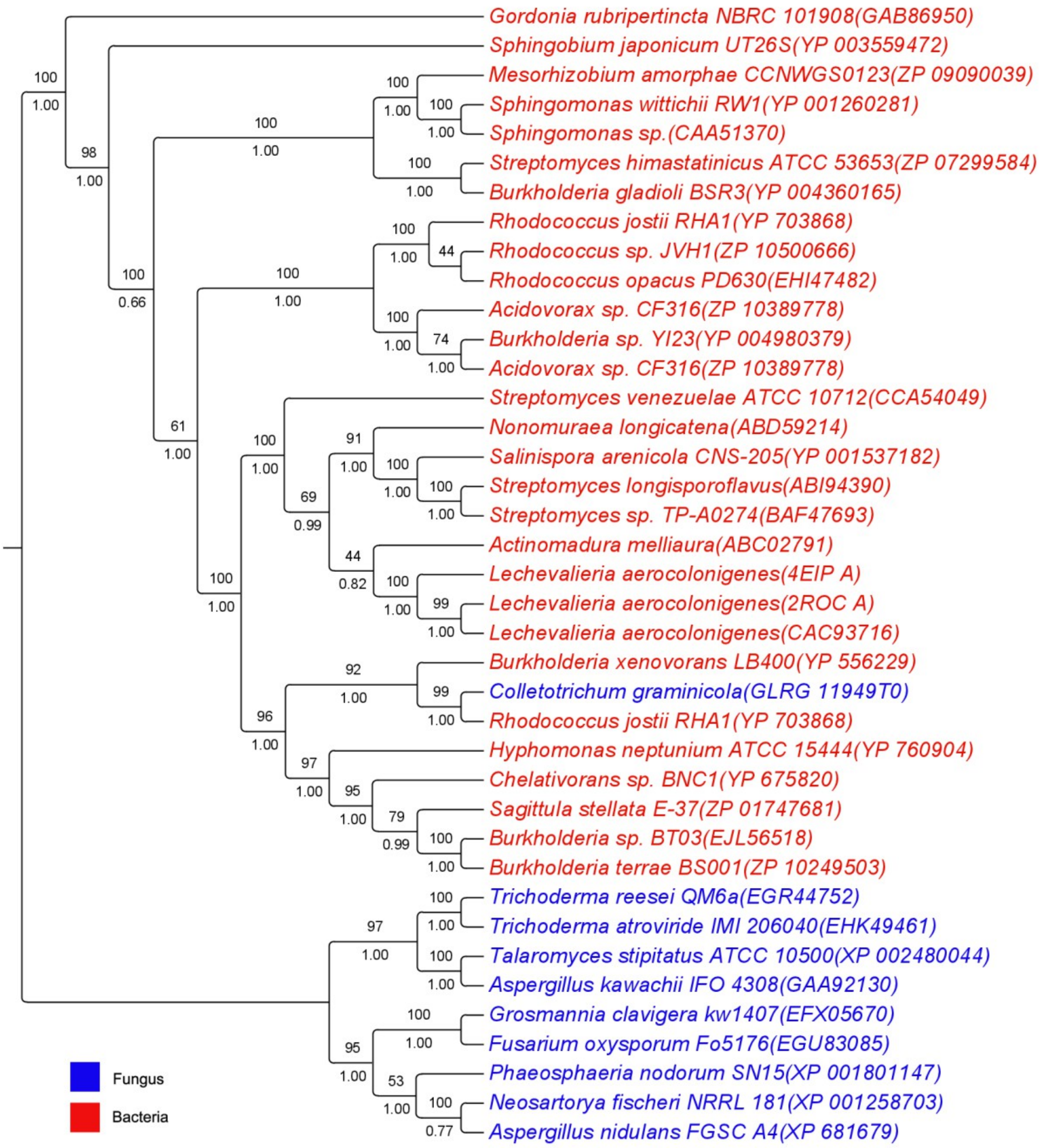

Figure S10. Maximum likelihood tree of HGT10 (GLRG_11949T0). Bootstrap percentage is shown above the branches and posterior probability is show below the branches. Accession numbers are shown in parenthesis next to the species names.

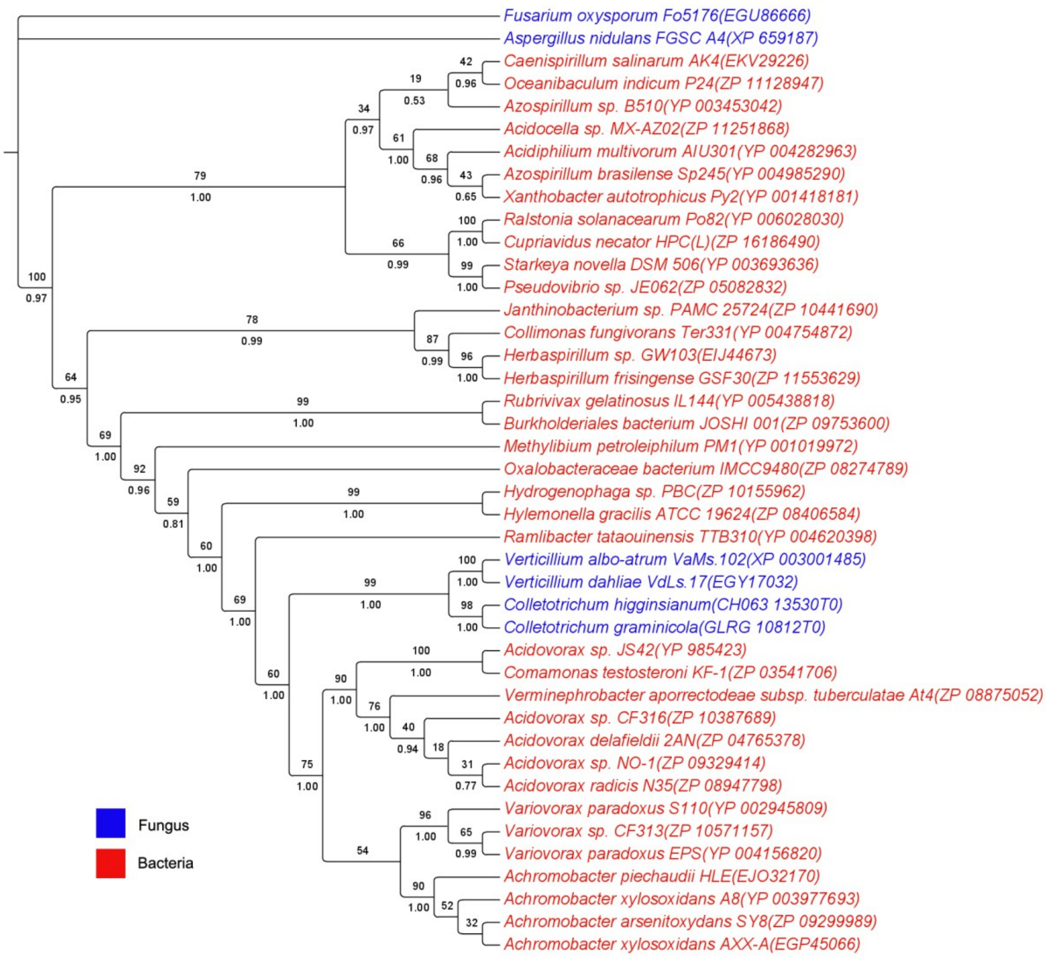

Figure S11. Maximum likelihood tree of HGT11 (GLRG_10812T0, CH063_13530T0). Bootstrap percentage is shown above the branches and posterior probability is shown below the branches. Accession numbers are shown in parenthesis next to the species names.

References

1. Ohm RA, Feau N, Henrissat B, Schoch CL, Horwitz BA, Barry KW, Condon BJ, Copeland AC, Dhillon B, Glaser F, Hesse CN, Kosti I, LaButti K, Lindquist EA, Lucas S, Salamov AA, Bradshaw RE, Ciuffetti L, Hamelin RC, Kema GHJ, Lawrence C, Scott JA, Spatafora JW, Turgeon BG, de Wit PJGM, Zhong S, Goodwin SB, Grigoriev IV: **Diverse lifestyles and strategies of plant pathogenesis encoded in the genomes of eighteen Dothideomycetes fungi**. *PLoS Pathog* 2012, **8**:e1003037.

2. Akinsanmi OA, Chakraborty S, Backhouse D, Simpfendorfer S: **Passage through alternative hosts changes the fitness of *Fusarium graminearum* and *Fusarium pseudograminearum***. *Environ Microbiol* 2007, **9**:512–520.

3. Freeman J, Ward E: ***Gaeumannomyces graminis*, the take-all fungus and its relatives**. *Mol Plant Pathol* 2004, **5**:235–252.

4. Gan P, Ikeda K, Irieda H, Narusaka M, O'Connell RJ, Narusaka Y, Takano Y, Kubo Y, Shirasu K: **Comparative genomic and transcriptomic analyses reveal the hemibiotrophic stage shift of *Colletotrichum* fungi**. *New Phytol* 2013, **197**:1236–1249.

5. Vargas WA, Martín JMS, Rech GE, Rivera LP, Benito EP, Díaz-Mínguez JM, Thon MR, Sukno SA: **Plant defense mechanisms are activated during biotrophic and necrotrophic development of *Colletotricum graminicola* in maize**. *Plant Physiol* 2012, **158**:1342–1358.

6. O'Connell RJ, Thon MR, Hacquard S, Amyotte SG, Kleemann J, Torres MF, Damm U, Buiate EA, Epstein L, Alkan N, Altmüller J, Alvarado-Balderrama L, Bauser CA, Becker C, Birren BW, Chen Z, Choi J, Crouch JA, Duvick JP, Farman MA, Gan P, Heiman D, Henrissat B, Howard RJ, Kabbage M, Koch C, Kracher B, Kubo Y, Law AD, Lebrun M-H, et al.: **Lifestyle transitions in plant pathogenic *Colletotrichum* fungi deciphered by genome and transcriptome analyses**. *Nat Genet* 2012, **44**:1060–1065.

7. Lowe RGT, Howlett BJ: **Indifferent, affectionate, or deceitful: lifestyles and secretomes of fungi**. *PLoS Pathog* 2012, **8**:e1002515.

8. Landschoot PJ, Jackson N: ***Magnaporthe poae* sp. nov., a hyphopodiate fungus with a *Phialophora* anamorph from grass roots in the United States**. *Mycol Res* 1989, **93**:59–62.

9. Shivas R., Tan Y.: **A taxonomic re-assessment of *Colletotrichum acutatum*, introducing *C. fioriniae* comb. et stat. nov. and *C. simmondsii* sp. nov.** *Fungal Divers* 2009, **39**:111–122.

10. Zhang G, Liang Y: **Improvement of fruiting body production in *Cordyceps militaris* by molecular assessment**. *Arch Microbiol* 2013, **195**:579–585.

11. Clarkson JM, Charnley AK: **New insights into the mechanisms of fungal pathogenesis in insects**. *Trends in Microbiology* 1996, **4**:197–203.

12. Rougeron A, Giraud S, Razafimandimby B, Meis JF, Bouchara J-P, Klaassen CHW: **Different colonization patterns of *Aspergillus terreus* in patients with cystic fibrosis**. *Clin Microbiol Infect* 2013.

13. Döğen A, Kaplan E, Ilkit M, de Hoog GS: **Massive contamination of *Exophiala dermatitidis* and *E. phaeomuriformis* in railway stations in subtropical Turkey**. *Mycopathologia* 2013, **175**:381–386.

14. Najafzadeh MJ, Dolatabadi S, Saradeghi Keisari M, Naseri A, Feng P, de Hoog GS: **Detection and identification of opportunistic *Exophiala* species using the rolling circle amplification of ribosomal internal transcribed spacers**. *J Microbiol Methods* 2013, **94**:338–342.
